# Comprehensive identification of sexually dimorphic genes in diverse cattle tissues using RNA-seq

**DOI:** 10.1186/s12864-016-2400-4

**Published:** 2016-01-27

**Authors:** Minseok Seo, Kelsey Caetano-Anolles, Sandra Rodriguez-Zas, Sojeong Ka, Jin Young Jeong, Sungkwon Park, Min Ji Kim, Whan-Gook Nho, Seoae Cho, Heebal Kim, Hyun-Jeong Lee

**Affiliations:** Interdisciplinary Program in Bioinformatics, Seoul National University, Kwan-ak St. 599, Kwan-ak Gu, Seoul, South Korea 151-741 Republic of Korea; CHO&KIM genomics, Main Bldg. #514, SNU Research Park, Seoul National University Mt.4-2, NakSeoungDae, Gwanakgu, Seoul, 151-919 Republic of Korea; Department of Animal Sciences, University of Illinois, Urbana, IL 61801 USA; Department of Agricultural Biotechnology, Animal Biotechnology Major, and Research Institute for Agriculture and Life Sciences, Seoul National University, Seoul, 151-921 Republic of Korea; Division of Animal Products R&D, National Institute of Animal science, #1500 Kongjwipatjwi-ro, Wansan-gu, Jeonju-si, Jeollabuk-do 565-851 Republic of Korea; Department of food science and technology, Sejong University, 98 Gun-Ja-Dong, Seoul, 143-747 Republic of Korea; Department of Swine & Poultry Science, National College of Agriculture and Fisheries, #1515 Kongjwipatjwi-ro, Wansan-gu, Jeonju-si, Jeollabuk-do 560-500 Republic of Korea

**Keywords:** RNA-seq, Sexual dimorphism, Tissue-specific gene expression, ANODEV

## Abstract

**Background:**

Molecular mechanisms associated with sexual dimorphism in cattle have not been well elucidated. Furthermore, as recent studies have implied that gene expression patterns are highly tissue specific, it is essential to investigate gene expression in a variety of tissues using RNA-seq. Here, we employed and compared two statistical methods, a simple two group test and Analysis of deviance (ANODEV), in order to investigate bovine sexually dimorphic genes in 40 RNA-seq samples distributed across two factors: sex and tissue.

**Results:**

As a result, we detected 752 sexually dimorphic genes across tissues from two statistical approaches and identified strong tissue-specific patterns of gene expression. Additionally, significantly detected sex-related genes shared between two mammal species (cattle and rat) were identified using qRT-PCR.

**Conclusions:**

Results of our analyses reveal that sexual dimorphism of metabolic tissues and pituitary gland in cattle involves various biological processes. Several differentially expressed genes between sexes in cattle and rat species are shared, but show tissue-specific patterns. Finally, we concluded that two distinct statistical approaches have their advantages and disadvantages in RNA-seq studies investigating multiple tissues.

**Electronic supplementary material:**

The online version of this article (doi:10.1186/s12864-016-2400-4) contains supplementary material, which is available to authorized users.

## Background

The molecular mechanisms underlying sexual dimorphism have only been partially elucidated. Gene expression analysis has been utilized to answer relevant questions through analysis of transcriptomic differences. While many of these studies have been performed on data from humans, rodents, and fruit flies, only a few studies have attempted to characterize sexual dimorphism in cattle [1, 2]. Additionally, most bovine transcriptomic research with the goal of identifying mechanisms of sexual dimorphism related to tissue growth and development has been performed exclusively on pre-implantation embryos; exploratory analyses on other tissues may provide further insight into these mechanisms.

Bovine beef and milk are important sources of nutrition for humans [3]. The quality and quantity of these nutrition sources have been shown to be affected by sex and the expression of sex determination genes in the food-producing animal [4, 5]. Investigation of sexual dimorphism in metabolic tissues such as liver, muscle and adipose tissue from cattle is crucial for both research and the food production industry. Results of previous microarray studies which investigated sex differences in metabolic tissues and brain in mouse revealed that biological pathways are highly distinct between males and females. Additionally, comparison of sex in multiple tissues revealed a tissue-specific pattern of gene expression [6]. Given these results, we expect to find clear sexual differences in cattle metabolism as well.

While the entire brain displays sexually dimorphic phenotypes, the hypothalamus-pituitary axis acts as one of the primary structures which controls sexual dimorphism in the central nervous system (CNS) as well as peripheral tissues. The pituitary gland, known as the “master gland” of the body, acts as the central endocrine regulator of metabolism, growth, and sexual maturation. In order to perform these functions, unique cell types in the anterior pituitary gland secrete polypeptide hormones such as growth hormone (GH) and gonadotropins, a family of protein hormones including luteinizing (LH) and follicle stimulating (FSH) hormones, by appropriately orchestrating signals from environmental and internal stimuli. Additionally, profound sex differences exist in hormonal regulation and responses of the pituitary gland to external stressors, which is why females display a higher vulnerability to various neuropsychiatric disorders [7, 8]. Hence, examination of sexually dimorphic gene expression patterns in the pituitary gland simultaneously with metabolic tissues will improve our understanding of sexual dimorphism in physiological and metabolic perspectives [9, 10]. Additionally, there is a lack of a species-specific research using transcriptome analysis on bovine pituitary-gland tissues to explore sexual dimorphism.

In recent years, the experimental design of transcriptomic studies to detect differentially expressed genes (DEGs) has become increasingly more sophisticated in terms of considering multiple factors, which is often required for simultaneous investigation of multiple tissues samples. While most microarray studies employ a two-way analysis of variance (ANOVA) [11, 12] for detecting DEGs from multifactor designed data, only a few RNA-seq based transcriptome analysis studies continue to be performed using a multi-factorial design. There are two reasons why limited RNA-seq studies with complex experimental designs are performed. Firstly, high RNA-seq prices make biological replication difficult. Secondly, there is an absence of analysis methods for detection of DEGs in multi-factorial designed RNA-seq data. While complex-structured RNA-seq data can be analysed using *R* or *SAS*, such approaches are often inadequate for handling normalization, assumption of distribution, etc. Fortunately, recent methodological advances [13, 14] have made it possible to perform multi-factorial analysis on RNA-seq data by using an analysis of deviance (ANODEV) model.

In the present study, we aimed to identify sexual dimorphic genes that contribute to bovine sexual dimorphism. Two statistical approaches were utilized for analyzing complex RNA-seq data from samples collected from several different tissues- liver, muscle, visceral adipose tissue and pituitary gland: 1) a simple two group comparison for detecting sexually dimorphic genes in each tissue (M1); and 2) an ANODEV based approach which simultaneously considers not only the effect of sex but also tissue type on the model (M2). Here we report advantages and disadvantages of these approaches for identification of sexually dimorphic genes in several tissues, as well as identified diverse mechanisms of bovine sexual dimorphism.

## Results

### Description of the RNA-seq analysis pipeline

For extraction of RNA-seq gene expression data, we employed Trimmomatic [15]. As shown in (Additional file 1: Table S1), this resulted in clean reads (adapter sequences removed) with a 98.75 % average surviving reads rate among 40 samples. These reads were aligned to the cattle reference genome (*bos taurus7* from the UCSC genome database) using Bowtie2 with default options with an average 81.91 % mapping rate. We calculated gene expression using General Transfer Format (GTF) file from UCSC genome browser with mapped result on HTSeq python package. 13,570 total genes resulted from this pipeline and detailed numbers of genes in each chromosome is reported in (Additional file 1: Table S2). Non-expressed genes across all samples were removed. A total of 13,148 genes were used for DEG analysis.

### Identification of sexual dimorphic genes using M1 in each tissue

**Fig. 1:**
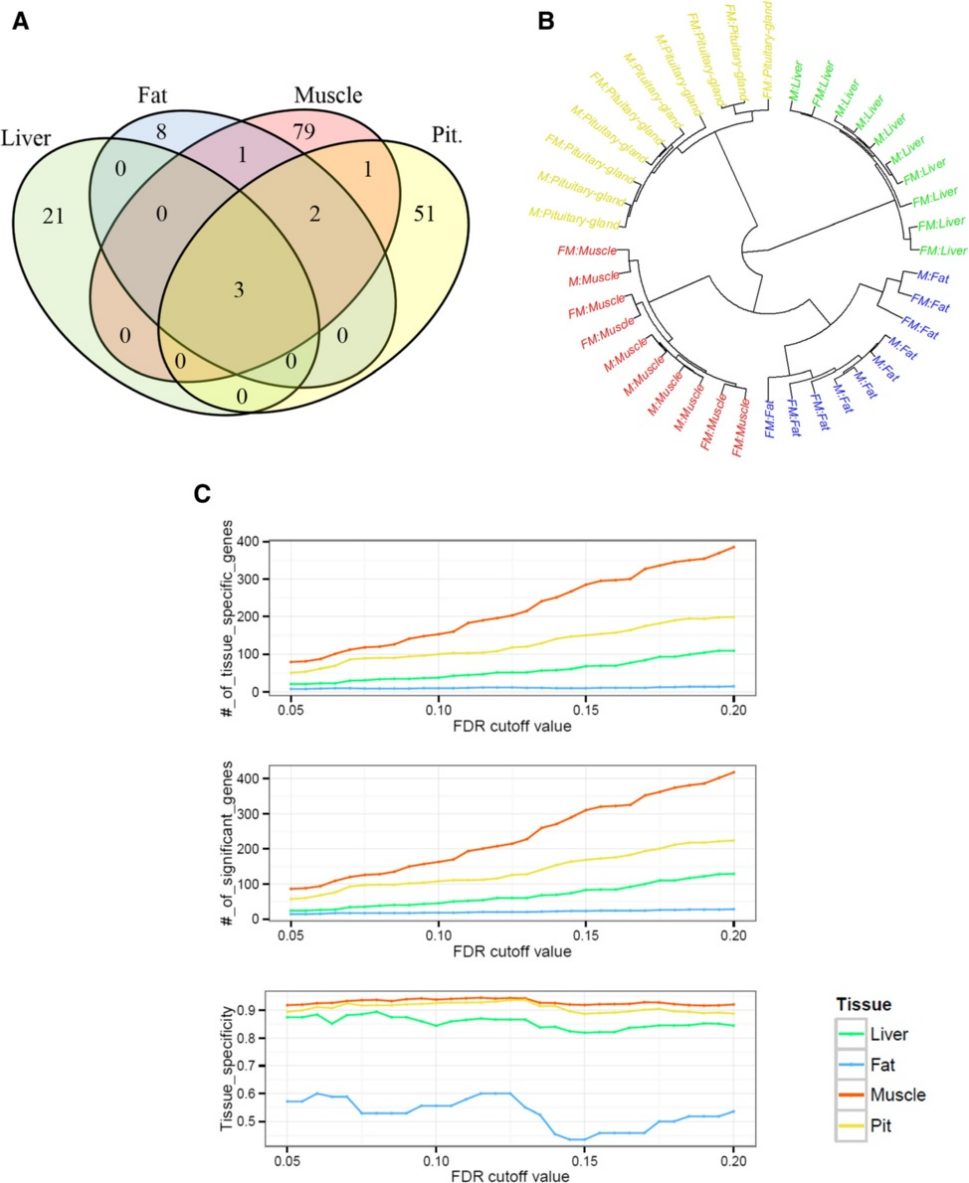
Tissue specificity of the detected sex-related genes. **a** Venn diagram of DEGs list using a two-group test in each tissue (FDR adjusted *P*-value < 0.05). **b** Result of hierarchical clustering among each tissue samples using the all genes with Pearson correlation coefficients. **c** Number of detected genes and tissue specific genes by FDR cutoff and their tissue specificity calculation

For identification of sexual dimorphic genes in several tissues, we performed a two-group test on each tissue using M1. As a result, 24, 14, 86, and 57 genes were detected as significantly differentially expressed in liver, fat, muscle, and pituitary-gland tissues, respectively (FDR adjusted *P*-value < 0.05). Three genes were commonly identified in all four tissues: *DDX3Y, USP9Y*, and *ZFY* (Fig. 1-(a)). Significantly detected DEGs reveal high tissue-specificity; only a few DEGs were found commonly significant in several tissues simultaneously. Of the 24 significantly detected DEGs in liver tissue, 21 genes were only identified in liver tissue, including *CUX2* (FDR adjusted *P*-value: 4.41E-04), *CYP7A1* (4.41E-04), *AK4* (1.05E-03), *COL27A1* (1.18E-03), and *TNC* (1.32E-03). This pattern of strong tissue-specificity in detected DEGs was also observed in other tissues. In fat tissue, 8 out of 14 total DEGs were identified as fat-tissue specific sexually dimorphic gene, including *IGFBP1* (2.02E-04), *TECTB* (6.77E-03), and *ACR* (1.24E-02). In muscle tissue, the highest number of sexual differenced genes was detected. While 86 genes were significantly detected, only 7 DEGs were commonly identified in other tissue. *MYH1* (7.51E-16), *MMP12* (1.02E-07), *MCHR1* (4.15E-05), and *SH3KBP1* (2.89E-04) were found to be extremely significant between female and male exclusively in muscle tissue. Finally, of 57 DEGs detected as significant in pituitary-gland tissue, 51 of those were tissue specific including *GRP* (1.79E-14), *LOC781146* (2.70E-09), and *LYSB* (9.12E-08). For more detailed investigation of tissue specificity, we performed hierarchical clustering in order to uncover the relationship among tissues using all genes. Figure 1-(b) shows male and female samples clustered within each tissue; results reveal that expression differences between sexes are less than the differences across tissues. In this figure, within group similarity of fat tissue samples is observed to be lower than the others. However, this phenomenon can not only be observed at a certain FDR cut-off; tissue-specificities were also calculated by adjusting FDR-adjusted *P*-value cutoff 0.05 to 0.2 as shown in Fig. 1-(c). Although this significance cutoff was varied (0.05 to 0.2), overall trends were maintained in terms of ranking of tissue specificity.

### Characterizing sexual dimorphic genes in relation to sex biasness, chromosomal location, and tissues

**Fig. 2:**
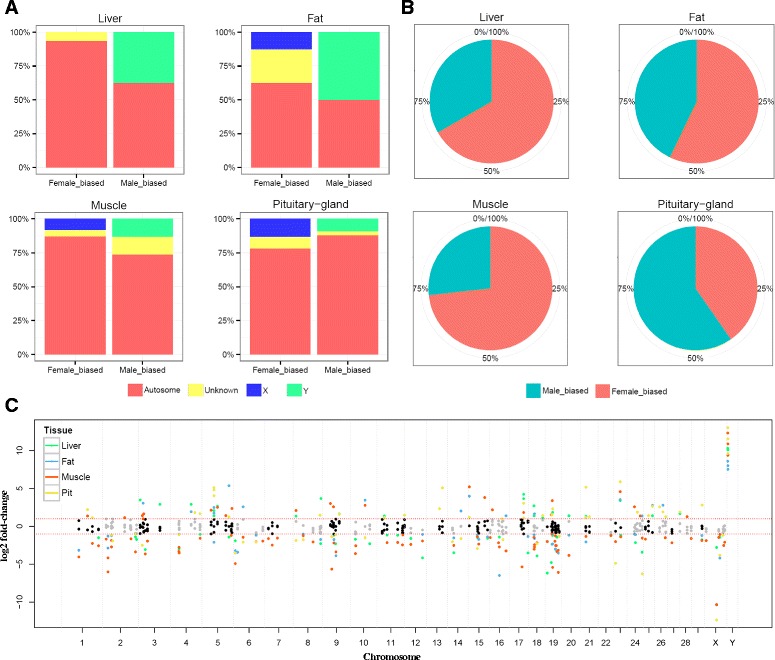
Distinguishing significant sexual dimorphic genes (FDR < 0.05) from several categories including sex biasness, tissues, and chromosomal location. **a** The box plot composed of two factors, sex biasness and chromosomal allocation, in each tissue **b** The pie charts showed proportion of male and female biased genes in each tissue, respectively **c** Manhattan plot for allocating sexual dimorphic genes from located chromosomal position with y-axis is a logged2 fold-changes (Male: Female). A total of 166 significantly detected DEGs using M1 across whole tissues were visualized. The red-dotted line represents the 2-fold change cutoff.

A previous sexual dimorphism study in mouse [6] defined over-expressed genes as sex-biased genes. Following this definition, sex biasness was investigated in our study in order to identify bovine sexual characteristics. Firstly, sexually dimorphic genes in each tissue detected using M1 were distinguished using both sex-biased information and chromosomal location such as autosome (1 ~ 29 chromosome), unknown, X, and Y chromosome which is based on gene annotation (Fig. 2-(a)). This figure reveals two primary differences among tissues in terms of proportion with significance level (FDR adjusted *P*-value < 0.05). First, all Y-chromosomal sexually dimorphic genes were observed to be male-biased. Second, no male-biased genes located in the X-chromosome were observed.

Figure 2-(B) shows significantly detected genes classified according to the proportion of sex biasness of each tissue. High female biasness was observed in liver (66.6 %), fat (57.14 %), and muscle tissues (73.25 %). On the other hand, a different pattern was observed in the pituitary-gland; the percentage of female biased genes in this tissue was lower than that of male biased genes (40.35 %). As this result could be only observed at a specific threshold value, we attempted to observe expression patterns by varying the FDR cutoff (0.05, 0.1, and 0.2) (Additional file 1: Figure S1). Although the proportion of female biased genes was higher than that of male biased genes with FDR adjusted *P*-value < 0.1 or <0.2 in pituitary-gland tissue, the relatively high proportion of the male biased genes is preserved when comparing to other tissues. Based on this observation, statistical tests were performed in order to examine this pattern of detected sexually dimorphic genes being enriched as male-biased genes in the pituitary-gland tissue more than in other tissues (Additional file 1: Table S3). Results revealed that only detected DEGs (FDR adjusted *P*-value < 0.05, 0.1, and 0.2) in pituitary-gland tissues were significantly male-biased.

Significantly detected sexually dimorphic genes were visualized as a transcriptional Manhattan plot (Fig. 2-(c)) [16]. From this type of plot, comprehensive patterns can be panoptically recognized using detected genes in each tissue with their chromosomal information and log2 fold change (logFC) between female and male expression. To visualize an RNA-seq version of Manhattan plot, we used gene location information from the transcriptome reference file. Unlike SNP markers, genes are not specific to one base position, so we considered the central position of the gene region as that gene’s chromosomal location in order to apply RNA-seq data. Detected sexually dimorphic genes were shown to spread all over the chromosome (see also Additional file 1: Table S4), however, extremely strong signals were identified in the sex chromosome (see also, Additional file 1: Figure S2, S3, and S4). These figures reveal that although sexual dimorphic genes are spread all over the chromosome, extremely large differenced genes based on the logFC can be identified in sex chromosome.

### Identification of sexual dimorphic genes considering the effect of whole tissue simultaneously using a statistical model based approach

In order to simultaneously consider the effect of tissue on the statistical model and identify sexually dimorphic genes, ANODEV implemented within *edgeR* [13] was used to allow for identification of DEGs in cases where the data structure is more complex. M2 was used for detection of sexual dimorphic genes when adjusting for the effect of tissue on the model. As a result, 655 significant (FDR adjusted *P*-value < 0.05) DEGs were identified using a likelihood ratio test (LRT) including Y-linked genes such as *USP9Y* (5.47E-72), *DDX3Y* (6.56E-55), and *ZFY* (8.79E-55), X-linked genes such as *XIST* (1.37E-17), *KDM6A* (9.01E-05), and autosomal genes such as *LOC780876* (3.71E-12), *LYSB* (5.71E-06), and etc. which appeared on the list of the top 20 most significant genes (Additional file 1: Table S5). We compared the number of significant genes using a Venn diagram, varying FDR-adjusted *P*-value cutoffs such as 0.05, 0.1, and 0.2 (Additional file 1: Figure S5). A larger number of sexually dimorphic genes were identified using M2 than using M1. In addition, approximately half of the significantly detected DEGs resulting from using M1 were shown to overlap with the integrated model (M2); the proportion of overlapped genes was 40 ~ 92 % (Additional file 1: Table S6). However, there were a large number of non-overlapped genes which were only significantly detected when using either M1 or M2. We examined potential factors causing this clear distinction between M1 and M2. Under a FDR adjusted *P*-value cutoff of 0.05, 752 genes were significantly detected using M1 and M2 (Additional file 2). Of these genes, three representative genes which showed distinctly different patterns between M1 and M2 were visualized as a box-plot (Additional file 1: Figure S6) and a line-plot (Additional file 1: Figure S7). It can be observed in these figures that significantly detected DEGs from M2 show relatively similar slopes among the tissues compared to significantly identified genes from M1. As shown in Figure S6-(A), *AK4*, *ANXA9*, and *STRA6* were selected as representative genes for M1. *AK4* (FDR adjusted *P*-value: 0.001 in the liver) was significantly detected in M1, but was not significantly detected in M2 (0.476). Likewise, *ANXA9* and *STRA6* genes were significant in M1 (0.049, 0.014) in muscle and pituitary-gland, respectively. However, M2 reported FDR adjusted *P*-values of 0.782 and 0.891 for muscle and pituitary-gland respectively. Contrastively, *AGPHD1*, *KDM6A*, and *SRSF2* genes were only significant using M2 (FDR adjusted *P*-values were 0.001, 7.99E-05, and 0.0007, respectively). M1 did not significantly detect any of these genes in any tissue.

### Chromosomal enrichment analysis using significant sexual dimorphic genes from M2

**Fig. 3:**
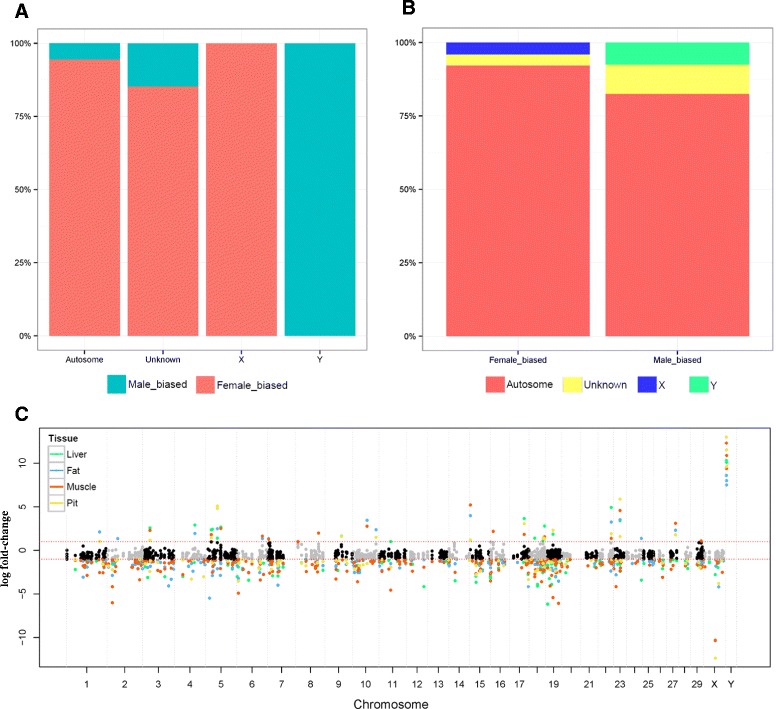
The summary of the pattern of sexual dimorphic genes resulting from M2 (FDR < 0.05). **a** Proportion of the male or female biased gene in chromosomal group such as autosome, Un_random, X, and Y chromosome. **b** Proportion of the detected sexually dimorphic genes based on chromosomal location in female and male biased, respectively. **c** Significantly detected 655 DEGs were visualized as Manhattan plot using log fold-changes. The red-dotted lines represent cutoff of 2-fold change

Sexually dimorphic genes are well known to not only be widespread across chromosomes, but also show high enrichment in the sex chromosome across diverse species [6]. Based on the 655 DEGs significantly detected using M2, chromosomal enrichment analysis was conducted in order to check the degree of chromosomal enrichment. To investigate the degree of enrichment in each chromosome of cattle species, a Fisher’s exact test was employed using the DEGs identified using M2. Chromosome 10, 18, X, and Y were shown to be significantly (*P* < 0.05) enriched (Table 1). As expected given results of previous studies, sexual dimorphic genes were highly enriched in the X and Y chromosome in cattle (See Additional file 1: Figure S8). This result implies that sexual dimorphic genes cover not only sex chromosome but autosome as well. Additionally, creation of a Manhattan plot revealed that M2 detected a larger number of DEGs than M1. Based on the detected 655 genes in M2, sex biasness was calculated using chromosomal information (Fig. 3-(a)). A high percentage of the detected genes were observed as female biased gene, except for those found in the Y chromosome. Understandably, detected sexual dimorphic genes located in sex chromosomes showed specific sex biased expression without any exception. In the autosome and unknown chromosome (Un_random), although a few male biased genes were observed, most detected genes were female biased genes. As shown in Fig. 3-(b), sexually dimorphic genes of autosome and unknown chromosome were identified in both sex biased categories. In contrast, X and Y linked genes were only identified in female and male biased categories, respectively. In this figure, it can be observed that this pattern is very similar with muscle tissue results using M1 (Fig. 2-(a)). In order to confirm this result, log fold-changes were visualized as a logFC based Manhattan plot (Fig. 3-(c)) for the significantly observed 655 detected genes. Additionally, their FDR adjusted *P*-values of M1 were visualized as adensity plot (Additional file 1: Figure S9). These plots reveal that a larger number of DEGs were observed in muscle tissue than in others, and most DEGs showed female-biased expression.

**Table 1:** Results of chromosomal enrichment test (Fisher’s exact test)

Chr.	*P*-value	Chr.	*P*-value	Chr	*P*-value
Chr. 1	0.432	Chr. 12	0.354	Chr. 23	0.546
Chr. 2	0.224	Chr. 13	0.997	Chr. 24	0.72
Chr. 3	0.839	Chr. 14	0.541	Chr. 25	0.958
Chr. 4	0.891	Chr. 15	0.893	Chr. 26	0.947
Chr. 5	0.573	Chr. 16	0.621	Chr. 27	0.527
Chr. 6	0.460	Chr. 17	0.705	Chr. 28	0.794
Chr. 7	0.593	Chr. 18	0.009*	Chr. 29	0.214
Chr. 8	0.611	Chr. 19	0.058	Chr. X	0.02*
Chr. 9	0.736	Chr. 20	0.927	Chr. Y	0.005*
Chr. 10	0.04*	Chr. 21	0.955		
Chr. 11	0.887	Chr. 22	0.112		

(*) significant genes at *P*-value < 0.05

### Gene-set enrichment analysis for identification of biological mechanism about sexual dimorphism using significant genes from M2

**Fig. 4:**
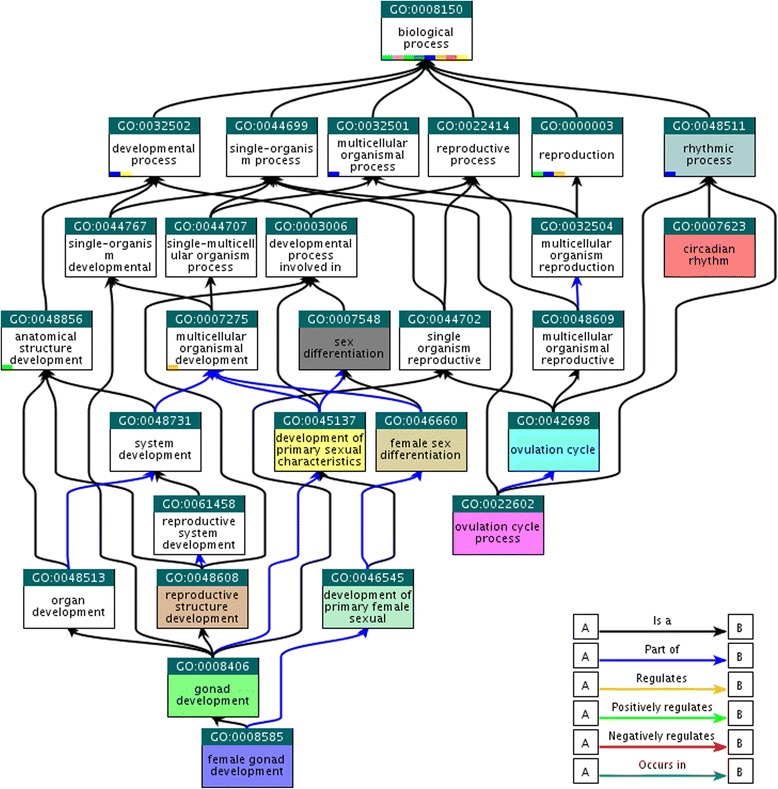
Diagram representing the relationship of significantly detected sex related cluster and their GO-terms by DAVID clustering analysis. Each box and line represents GO-terms and relationships, respectively. The figure was generated by QuickGO [59] with our detected biological terms using DAVID. In this diagram, coloring boxes are significant in our analysis

In order to better understand the biological processes related to sexual dimorphism, enrichment analysis was performed on the 655 DEGs detected using M2. DAVID analysis revealed 56 significantly enriched GO biological process terms (*P*-value < 0.05) and 11 KEGG pathways (Additional file 1: Table S7). Results of enrichment analysis revealed many different biological processes, presumably involved in cattle sexual dimorphism. Several immune system related terms were found enriched: *immune system development* (8.40E-02), *autoimmune thyroid disease* (3.90E-02), *T cell receptor signaling pathway* (9.60E-02), and *Natural killer cell mediated cytotoxicity* (9.60E-02). Additionally, several metabolic pathways between sexes such as *regulation of RNA metabolic process* (2.90E-04), *fructose and mannose metabolism* (1.20E-02), *glycosphingolipid biosynthesis* (6.50E-02) and *steroid hormone biosynthesis* (9.90E-02) were significantly identified. Finally, *calcium signaling pathway* (3.70E-03), *eye development* (6.30E-02) and *taste transduction* (7.60E-02) were found to be enriched as well. In order to segregate these diverse significant gene-sets, DAVID annotation clustering was performed and 14 enriched biological clusters exhibiting diverse biological activities were identified (Additional file 1: Table S8). Among them, one cluster containing several sex related terms was observed, which included *rhythmic process* (6.90E-04) and *circadian rhythm* (4.90E-02). In order to visually represent this relationship, a hierarchical relationship plot of GO terms was constructed (Fig. 4). This plot revealed that results appear to be nested underneath two representative groups: sex differentiation related and rhythmic process related terms. Several DEGs were associated with these terms, including *AFP*, *CGA*, *FOXL2*, *STAT5A* and *ANG2*. Five significant transcription factors were found to be associated with circadian rhythm: *PER2*, *NR1D1*, *DBP*, *NFIL3* and *BHLHE41*. They are known to participate in core transcriptional regulation of circadian rhythm and, according to our results, they may have roles linking circadian rhythm and/or metabolism in sexual dimorphism. These results have implicated various biological functions, including circadian rhythm, in sexual dimorphism in cattle.

### Technical validation to detect sexually dimorphic genes in two different species using qRT-PCR

**Fig. 5:**
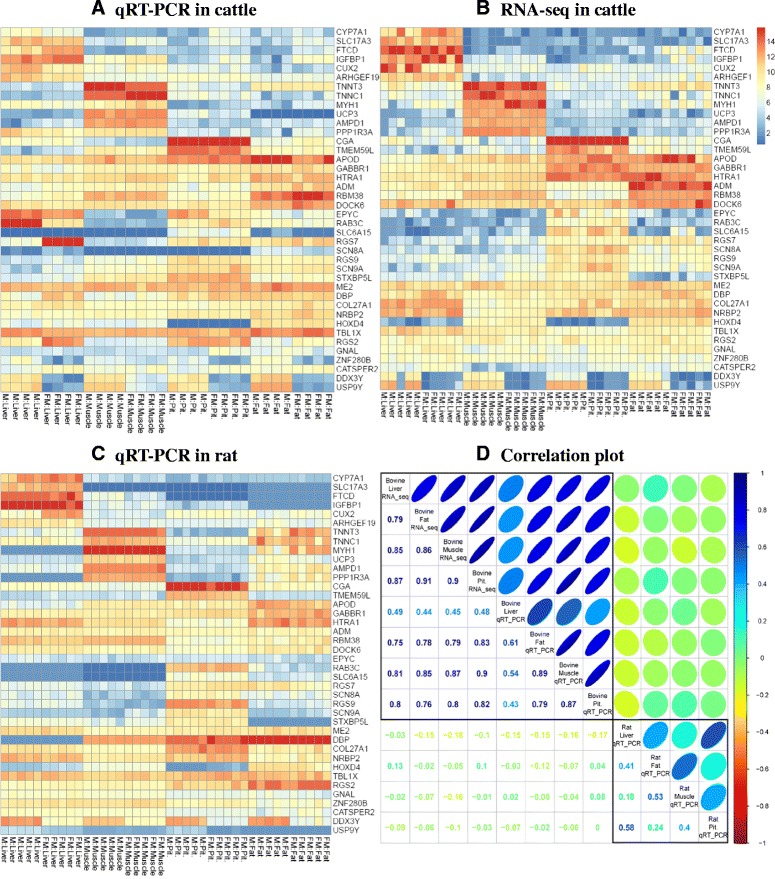
Relative Heatmap drawn using 40 randomly selected sexual dimorphic genes from cattle RNA-seq analysis. The intensities included in the relative Heatmap represent quantile-normalized values. **a** qRT-PCR result using 32 biological replicated samples for cattle species **b** gene expression from the RNA-seq in cattle species **c** qRT-PCR result using 40 biological replicated samples in rat species. **d** Quantification of the similarities among three experiments. Pearson’s correlation coefficients were employed with logged2 fold-changes (Male:Female) in each experiment

Technical validation was performed using qRT-PCR in order to 1) technically validate detected sexual dimorphic genes by comparing expressed genes between RNA-seq and qRT-PCR in cattle; and 2) compare cattle and rat to investigate whether validated sexually dimorphic genes are shared between these two different mammal species. Using two statistical approaches, M1 and M2, we identified 752 significant (FDR < .05) sexually dimorphic genes. Of these detected DEGs, 40 genes were randomly selected from the known primer sequence in cattle (Additional file 1: Table S9) and rat (Additional file 1: Table S10) species and qRT-PCR was performed in order to compare not only RNA-seq vs. qRT-PCR in cattle, but also cattle vs. rat. To examine global gene expression among three experiments, a comparative heatmap was visualized using quantile-normalized values (Fig. 5). First, most gene expression patterns were observed to be analogous between qRT-PCR and RNA-seq (Fig. 5-(a, b)). In order to quantitatively assess this observation, Pearson’s correlation coefficients were calculated using the log-fold change ratio between male and female among the three experiments as shown in Fig. 5-(d). As a result, high correlation coefficients (0.43 to 0.87) were observed in bovine RNA-seq and qRT-PCR. On the other hand, small correlation coefficients (-0.18 to 0.13) were observed between cattle and rat. In order to compare this validation result in another aspect, statistical tests were performed on our qRT-PCR results (Table 2). As the 40 sexually dimorphic genes were derived from RNA-seq analysis using a generalized linear model (GLM) such as M1 (two group comparison) and M2 (using ANODEV), a *t*-test and analysis of variance (ANOVA) were employed to apply similar statistical tests for qRT-PCR analysis. Most detected sexually dimorphic genes were significantly found in cattle and rat species using qRT-PCR. This statistical result reveals that although gene-expression patterns of the two species are not identical, sexually dimorphic genes can be commonly identified in both species. To examine this phenomenon, hierarchical clustering analysis was performed using gene expression data from the 40 genes using the *cluster* R package. Euclidean distance was employed for determining the distance among samples and calculating representative distance in each branch of the cluster. In addition, silhouette scores were calculated for detecting the optimal number of clusters for each of the three types of datasets. As a result, the optimal number of clusters, *k*, was estimated as 4 for RNA-seq on cattle and qRT-PCR on rat, but was estimated as 7 for qRT-PCR on cattle. Based on these estimated optimal number of clusters, hierarchical cut-trees were visualized for each dataset (Additional file 1: Figure S10). The three trees reveal not only that samples can be clearly distinguished by tissue, but also that samples are clearly divided based on sex in each tissue. These technically validated 40 selected genes were revealed to be sexually dimorphic genes in both cattle and rat species. Finally, of 40 randomly selected DEGs, 33 were significantly detected in M2. In order to measure the accuracy of the M2, statistical results of qRT-PCR and RNA-seq were compared (Table 3). As a result, 21 genes were technically validated in qRT-PCR (ANOVA analysis with a significance cutoff of 5 %).

**Table 2:** Statistical significance of qRT-PCR results for the selected set of 40 genes analyzed

Gene_symbol	Liver	Muscle	Pit.	Fat	ANOVA	Liver	Muscle	Pit.	Fat	ANOVA
ADM	7.30E-02	2.30E-01	6.90E-01	4.70E-01	2.70E-01	1.50E-01	3.00E-01	2.90E-03*	3.50E-03*	5.70E-01
AMPD1	8.50E-01	9.20E-02	4.00E-01	1.40E-01	2.40E-01	1.70E-01	1.30E-01	2.40E-02*	3.00E-01	4.20E-01
APOD	2.60E-02*	1.20E-01	3.40E-01	2.80E-02*	5.00E-02*	7.70E-02	2.30E-01	5.50E-01	6.20E-01	5.60E-02
ARHGEF19	2.90E-02*	1.60E-02*	6.90E-02	5.10E-03*	2.30E-03*	2.80E-01	2.50E-02*	4.70E-01	2.40E-01	4.60E-02*
CATSPER2	8.40E-03*	4.50E-02*	1.00E + 00	1.10E-01	6.50E-03*	6.30E-02	3.10E-01	5.50E-02	8.90E-01	9.30E-01
CGA	1.10E-02*	5.00E-03*	7.00E-02	5.50E-02	1.60E-01	5.40E-02	1.60E-01	2.20E-01	8.30E-04*	1.60E-01
COL27A1	1.90E-01	2.80E-02*	1.20E-02*	9.70E-03*	1.50E-02*	2.30E-01	1.50E-02*	6.00E-01	4.20E-01	9.00E-01
CUX2	6.10E-01	8.90E-04*	8.10E-01	4.20E-01	1.10E-02*	1.70E-02*	5.40E-05*	4.60E-02*	1.30E-01	9.40E-03*
CYP7A1	5.50E-01	1.50E-03*	2.50E-01	9.30E-03*	4.10E-01	4.40E-01	1.50E-01	1.10E-01	5.00E-01	9.50E-01
DBP	5.40E-02	2.40E-04*	4.70E-01	1.60E-01	3.40E-03*	2.90E-01	3.70E-01	2.60E-01	4.50E-02*	7.00E-01
DDX3Y	4.90E-08*	4.40E-06*	2.20E-03*	2.70E-06*	9.90E-22*	6.80E-04*	4.30E-02*	1.00E-05*	2.20E-08*	1.10E-14*
DOCK6	4.60E-02*	5.60E-02	6.90E-02	8.80E-01	6.00E-02	2.40E-01	4.70E-01	3.90E-02*	5.60E-01	1.10E-01
EPYC	5.80E-01	5.50E-01	3.90E-03*	1.50E-04*	1.30E-04*	1.90E-02*	1.50E-02*	9.70E-01	5.50E-02	7.70E-02
FTCD	2.30E-02*	8.40E-05*	3.40E-02*	4.90E-01	5.90E-05*	NA	3.70E-01	2.30E-03*	NA	7.60E-01
GABBR1	5.40E-01	2.70E-02*	4.60E-02*	6.50E-03*	6.10E-01	6.80E-02	4.90E-01	9.10E-01	2.40E-01	1.90E-01
GNAL	7.00E-01	5.70E-01	1.00E-01	4.20E-03*	3.00E-01	3.20E-01	4.30E-01	6.10E-01	4.80E-01	6.00E-01
HOXD4	8.50E-01	5.70E-01	8.30E-01	NA	5.10E-01	3.30E-01	1.30E-02*	4.30E-01	1.20E-01	1.60E-02*
HTRA1	4.60E-02*	5.80E-01	2.00E-01	3.70E-02*	5.50E-01	4.80E-01	4.20E-01	4.90E-02*	2.30E-01	3.30E-01
IGFBP1	1.90E-03*	8.10E-03*	7.50E-03*	1.00E-02*	6.70E-10*	NA	2.80E-01	4.20E-02*	8.40E-02	3.20E-01
ME2	1.40E-01	3.10E-01	2.90E-01	6.70E-01	7.80E-02	6.60E-01	4.60E-01	1.70E-01	1.30E-01	4.00E-01
MYH1	9.80E-02	4.20E-03*	1.70E-03*	1.30E-03*	2.90E-02*	1.80E-02*	NA	3.10E-01	1.50E-02*	1.40E-01
NRBP2	4.40E-01	1.20E-01	9.40E-03*	9.10E-01	1.80E-02*	3.80E-02*	3.80E-01	3.60E-02*	2.10E-01	1.20E-01
PPP1R3A	1.00E-01	5.40E-03*	6.00E-01	7.00E-01	4.80E-01	4.80E-02*	3.70E-01	8.40E-02	4.40E-01	4.70E-02*
RAB3C	2.30E-02*	4.60E-05*	7.30E-01	6.10E-03*	2.20E-02*	1.00E-02*	3.70E-01	NA	6.10E-01	8.40E-02
RBM38	3.90E-02*	5.60E-03*	6.60E-01	2.30E-02*	5.60E-04*	1.90E-02*	5.00E-01	2.20E-03*	8.40E-03*	2.30E-01
RGS2	2.70E-02*	6.40E-05*	6.70E-01	6.20E-01	1.60E-03*	2.30E-02*	3.40E-01	7.20E-01	4.30E-01	1.70E-01
RGS7	3.40E-01	1.00E-06*	8.40E-01	8.30E-01	5.20E-03*	3.40E-02*	4.90E-01	7.50E-01	7.70E-02	6.90E-01
RGS9	9.20E-01	5.60E-01	3.70E-01	2.10E-01	5.80E-01	2.00E-01	4.40E-01	4.60E-02*	9.10E-03*	6.80E-01
SCN8A	5.60E-02	2.50E-01	NA	NA	7.30E-01	5.10E-01	4.10E-01	9.80E-01	3.40E-02*	5.50E-01
SCN9A	3.80E-02*	1.80E-02*	7.00E-01	7.50E-01	1.50E-02*	5.90E-03*	4.50E-01	5.80E-03*	6.80E-04*	4.40E-02*
SLC17A3	1.60E-02*	5.20E-01	1.00E-02*	4.60E-02*	4.40E-05*	NA	3.40E-01	NA	NA	2.90E-01
SLC6A15	5.90E-02	NA	NA	9.80E-01	9.30E-02	3.80E-01	3.70E-01	NA	3.90E-02*	3.80E-01
STXBP5L	4.10E-02*	2.70E-01	8.90E-01	1.50E-01	2.90E-01	NA	5.80E-01	6.10E-01	6.80E-02	6.00E-01
TBL1X	6.40E-01	2.80E-02*	9.00E-01	4.20E-02*	8.10E-03*	6.00E-01	3.80E-01	7.20E-02	8.90E-01	2.80E-01
TMEM59L	7.80E-01	9.80E-02	5.80E-02	2.70E-02*	9.50E-02	4.40E-02*	3.10E-01	4.50E-01	1.50E-02*	5.30E-01
TNNC1	8.60E-01	4.10E-01	4.90E-02*	1.50E-01	1.00E + 00	6.50E-01	4.40E-01	2.50E-03*	8.30E-01	6.40E-01
TNNT3	1.50E-02*	6.40E-01	3.50E-02*	6.90E-02	1.20E-03*	2.10E-02*	3.20E-01	3.90E-02*	6.10E-02	1.80E-01
UCP3	NA	3.60E-01	9.80E-01	1.00E-01	8.10E-02	4.40E-02*	5.10E-01	6.80E-01	1.30E-02*	6.00E-01
USP9Y	5.70E-06*	2.70E-05*	8.20E-04*	7.90E-05*	2.30E-20*	NA	9.30E-01	5.70E-01	6.50E-04*	4.10E-01
ZNF280B	5.00E-03*	9.40E-05*	2.90E-03*	1.10E-03*	4.00E-12*	1.60E-01	3.60E-01	3.30E-02*	3.50E-01	2.20E-01
Experiment	qRT-PCR in cattle					qRT-PCR in rat				

(*) significant genes at *P*-value < 0.05

*NA* value represents non-measured expression in the qRT-PCR

**Table 3:** Comparison between qRT-PCR and RNA-seq results for the 33 sexually dimorphic genes significantly detected using M2

Gene_symbol	qRT-PCR	RNA-SEQ
ADM	2.7E-01	8.31E-03*
AMPD1	2.4E-01	4.14E-04*
APOD	5.0E-02*	1.62E-03*
ARHGEF19	2.3E-03*	3.50E-02*
CATSPER2	6.5E-03*	3.86E-03*
CGA	1.6E-01	3.27E-02*
COL27A1	1.5E-02*	2.40E-04*
CYP7A1	4.1E-01	7.40E-05*
DBP	3.4E-03*	1.12E-03*
DDX3Y	9.9E-22*	3.01E-50*
DOCK6	6.0E-02*	4.14E-04*
EPYC	1.3E-04*	1.35E-02*
FTCD	5.9E-05*	3.96E-02*
GABBR1	6.1E-01	2.76E-05*
GNAL	3.0E-01	5.57E-04*
HOXD4	5.1E-01	1.23E-02*
IGFBP1	6.7E-10*	2.49E-03*
MYH1	2.9E-02*	2.98E-02*
NRBP2	1.8E-02*	2.21E-04*
PPP1R3A	4.8E-01	2.42E-02*
RAB3C	2.2E-02*	1.46E-02*
RGS2	1.6E-03*	3.36E-02*
RGS7	5.2E-03*	4.18E-03*
RGS9	5.8E-01	4.14E-04*
SCN8A	7.3E-01	8.69E-04*
SCN9A	1.5E-02*	5.71E-03*
SLC17A3	4.4E-05*	4.09E-02*
SLC6A15	9.3E-02*	4.76E-02*
STXBP5L	2.9E-01	1.75E-02*
TMEM59L	9.5E-02*	2.71E-03*
TNNC1	1.0E + 00	1.81E-02*
UCP3	8.1E-02*	7.81E-03*
USP9Y	2.3E-20*	5.20E-70*

(*) significant genes at *P*-value < 0.05 for ANOVA analysis in qRT-PCR and FDR adjusted *P*-value < 0.05 for ANODEV analysis in RNA-seq

## Discussion

### Bovine sex-chromosomal genes detected from RNA-seq analysis

As we excluded genes with very low expression levels (non-expressed genes in all samples) in this study, we expected all Y-linked genes to exhibit significant male-biased expression. However, only a few Y-linked non-DEGs were identified as a result of our analyses. Of 8 annotated genes based on the *bosTau7* reference genome, five genes (*EIF1AY, PRAME, SRY, TBL1X*, *ZNF280B)* were not significantly detected in M1 and M2. Three genes (*EIF1AY*, *PRAME*, *SRY*) had extremely low TMM normalized values. *TBL1X* and *ZNF280B* showed a large TMM normalized value (4.04 and 2.53, respectively) (Additional file 1: Figure S11). To investigate the possibility of cross-mapping between each of the gene and its paralogues, local alignment was performed using the *ZNF280B* mRNA-sequence to the reference genome using a basic local alignment search tool (BLAST). As a result, we not only observed the *ZNF280B* gene with 98 % query cover rate, but also expression of an un-annotated gene located in chromosome 17 with a 92 % query cover rate. In addition, a previous study identified *ZNF280B* as a autosome derived Y-chromosome gene [17]. Therefore, appearance of Y-linked non-DEGs may be attributed to their low expression or to variable between replicates to be found differentially expressed, or could be caused by two genes in the reference gene with similar sequences.

We observed no male-biased genes on the X chromosome at FDR adjusted *P*-value < 0.05 (Additional file 2). However, three X-linked genes were significantly up-regulated in males at FDR adjusted *P*-value < 0.1 (Additional file 1: Figure S12; see also Additional file 2). Three genes; *ZIC3* (FDR adjusted *P*-value: 0.083 in M2), *CXHXorf34* (0.095), and *TMEM35* (0.097) showed relatively little difference in expression between females and males, but also high individual variations compared to the other 752 significantly observed sexually dimorphic genes (FDR adjusted *P*-value < 0.05). Of the 325 X-chromosomal annotated genes, 26 were female-biased DEGs detected in either M1 or M2. These genes may serve a good candidates for X-chromosome inactivation escaping gene in cattle species (XCI-escaping gene). In muscle tissue, five such genes were identified: *CA5B* (4.97E-02), *RBM3* (4.34E-02), *SH3KBP1* (2.88E-04), *XPNPEP2* (4.69E-02), and *XIST* (5.83E-92). Additionally, *NXF3* (1.35E-03), *XIST* (4.25E-124), and *ZFX* (1.45E-02) were detected as DEGs in pituitary-gland tissue.

Of these genes, only one X-linked gene, *XIST*, was commonly identified in several tissues. *XIST* was significantly detected using M1(3.06E-47, 5.87E-92, and 4.26E-124 in fat, muscle, and pituitary-gland tissue, respectively) and M2 (1.34E-16). However, XIST was not significantly detected in liver tissue; this result was surprising, as we expected this female-specific X-inactivation related gene to be included in whole tissues. Examination of raw data revealed expression outliers in the liver in both female and male (Additional file 1: Figure S13), which resulted in no statistical significance (FDR adjusted *P*-value 0.591) and eventually eliminated *XIST* from the DEG list.

### Sexual dimorphism displayed in metabolic tissues and pituitary gland

We investigated causes for why male biased genes were enriched only in pituitary-gland tissue. Numerous muscle development related gene-sets were significantly reported by DAVID including *striated muscle tissue development* (1.30E-03), *muscle tissue development* (1.60E-03), *cardiac muscle tissue morphogenesis* (1.10E-02), *muscle tissue morphogenesis* (1.10E-02), *cardiac muscle tissue development* (5.10E-02), *skeletal muscle tissue development* (7.40E-02), and *skeletal muscle organ development* (7.40E-02) among others (Additional file 1: Table S14).. Results of previous studies [18], have shown that androgens play a highly important role in the development of muscle and bone, particularly in males. It appears that significantly detected sexual dimorphic genes in pituitary-gland tissue show male-biased enrichment given that many of the genes found significant in this tissue play an important role in muscle development. The results revealed that the effect of muscle tissue on about sex differences in gene expression is higher than that of other tissues. These results are consistent with earlier findings from studies using M1. These results and the number of detected genes imply that gene expression in muscle tissues is more impacted by sexually dimorphism than other tissues.

The pituitary gland is a critical structure for rhythmic control of metabolism and reproduction; this function is the result of hormones such as growth hormone (GH) and the gonadotropins being secreted in a pulsatile manner. Although the relationship among sexual dimorphism, metabolism and rhythmic process is complicated and not well-understood, identification of significant rhythmic process-related genes and pathways might indicate physiological roles of the pituitary gland and its effects on peripheral tissues. For instance, it is well recognized that a large number of sex differences in liver gene expression are controlled by the central circadian system [19–22] as well as result from the pulsatility of circulating GH [23, 24]. However, a non-circadian role for core circadian oscillators cannot be excluded [25, 26], and the circadian rhythm-related genes may be DE regulated downstream of these signaling cascades. Taken together, results of this study suggest that pituitary gland-expressed genes might at least be partly involved in the cascade of events starting with secretion of hormone, gene expression in peripheral tissues and, finally, establishment of sexual dimorphism in metabolic processes.

### Degree of sexual dimorphism in abdominal fat tissue gene expression

Results from RNA-seq analysis performed using M1 revealed that DEGs detected from abdominal fat tissue not only showed very little tissue specificity compared to liver, muscle, and pituitary gland, but also the lowest number of detected sexual dimorphic genes. This suggests that most of the genes found significant in fat tissue have a smaller effect on sexual dimorphism than the other tissues. Sex biased differences in fatty acid metabolism and regional fat distribution have been well recognized in previous studies [27, 28]. However, the number of studies on sex differences in gene expression of abdominal fat is limited and insufficient to elucidate the degree of sexual dimorphism in fat tissue. Meanwhile, several studies have reported that some aspects of visceral adipose tissue function appear to act independent of sex. For example, a previous study reported that no sex differences in androgen binding [29] and estrogen receptor expression [30] can be observed in human adipose tissue. Furthermore, another possible reason for the detection of fewer DEGs in fat tissue than in other tissues could be the breeding strategy for Korean native cattle, which was the species studied in the present study. Because highly marbled beef is preferred by consumers and manufacturers in the Korean beef industry, the cattle breeding system is mainly focused on increasing fat marbling [31, 32], which most likely has caused a gradual decrease in gene expression differences between female and male. Further investigation at the transcriptomic, proteomic and physiological levels should be performed to conclude whether fat depots possess a lesser extent of sexual dimorphism compared to other metabolic tissues.

We observed a large number of pituitary gland up-regulated in males, while the majority of liver, fat and muscle DEGs were up-regulated in females. There have been several previous studies that compared sexual dimorphism at a gene expression level between tissues, including the pituitary gland [9, 33]. While these results do not explain why pituitary gland genes appear to show sex-specific expression patterns, it may be beneficial for future studies to identify whether male-biased gene expression pattern in the pituitary gland appear in other species such as human and rodents.

### Comparison of two statistical approaches for detecting sexual dimorphic genes using two-way factorial designed data

In this paper, we used two statistical approaches, M1 and M2, to detect sexually dimorphic genes from two-way factorial RNA-seq data. M1, the simplest method for discovering DEGs, involves performing two group comparisons between female and male in each tissue. From this approach, a list of sexual dimorphic genes was for each individual tissue. Using these results, tissue specific sexually dimorphic genes (identified in only one tissue) or commonly identified genes in several tissues was identified by comparing lists of significant genes. Although our RNA-seq experiment produced 40 RNA-seq samples from 4 tissues, the M1 approach used only 5 (female) vs. 5 (male) samples from each tissue. Due to this small sample size, a relatively smaller number of DEGs were detected by M1 compared to M2; this issue of sample size leads to a loss of statistical power when using this model. This problem is widely discussed in microarray analysis [34–36]; comparison between *t*-test and contrast ANOVA reveals that contrast based methods are more statistically powerful than a simple two group test, since a contrast based approach makes it possible for more elaborate variance estimation given the larger number of samples used. Additionally, estimation of the interaction effect between two factors is only possible using an integrated model. Given these advantages, an ANOVA type model on GLM, the ANODEV, has been developed for RNA-seq data analysis. By employing ANODEV, we can consider more complex structured experimental design on the statistical model [13]. By considering several factors on the model, more DEGs can be significantly detected by increasing sample size. As we had expected, when applying these two statistical approaches for detection of sexual dimorphic genes, different patterns of detected DEGs were observed (Additional file 1: Figure S6 and S7). This difference is mainly due to the different statistical assumptions of each approach. In short, while M1 is suitable for finding tissue-specific sexually dimorphic genes, its statistical power is lower than the integrated model, which leads to a high rate of false negatives. In contrast, while M2 tends to detect more significant genes than M1, it has a relatively higher rate of false positives. We observed a 0.36 false positive rate (12 / 33) based on qRT-PCR validation results; 33 genes were significantly identified in RNA-seq analysis with M2, while 12 genes were found not significant in the qRT-PCR result (Table 3). This highly false positive rate may be attributed to a broken equal variance assumption. M2 assumes equal variance of each individual, however, highly tissue-specific patterns of gene expression make it difficult to precisely detect DEGs. This result suggests that equal variance can not be guaranteed when employing several tissues in RNA-seq analysis. Furthermore, in general RNA-seq experiments where testing on multiple tissues was performed, tissue samples were extracted from one individual, which leads to a complex correlated structure; this is referred to as a nested design [37]. One solution, which has already been suggested in microarray studies [38], is to estimate coefficients in the model using the weighted least squares (WLSE) method, which would allow for consideration of complex correlation structure. In addition, a generalized estimating equation (GEE) model could also be applied for repeated measure RNA-seq data analysis [39]. Although an ANODEV model implemented in edgeR and *DESeq2* [40] is able to consider this nested design by adding fixed-effect dummy variables for distinguishing nested variables, this is merely a second-best solution. Development of new methodology would allow for more accurate estimation using samples taken from several tissues. Another inconvenience of M2 is that we cannot distinguish the effect of specific tissues because post hoc analysis is not yet provided for RNA-seq studies. This issue has been widely discussed in regards to microarray data analysis on multilevel dataset [41, 42]. In short, by performing post-hoc test on each tissue (i.e. Tukey’s test in ANOVA) after the testing main factor (i.e. sex term on the M2), we are able to determine which tissue’s effect causes a sex difference. Unfortunately, as far as we know, no known attempts have been made to develop post-hoc test in RNA-seq analysis so far. More research is required to develop accurate methods for analysis multi-factor designed data using an integrated model based approach.

While statistical tests were performed using *edgeR* in this study, *DESeq2* also provides ANODEV. The list of DEGs resulting from an analysis could be highly influenced by several factors including choice of aligner, counter, statistical tools, and specific parameters. Unfortunately, it is nearly impossible to determine the optimal pipeline for each individual study given the high number of possible combinations of those factors. In this study, we mainly focused on the comparing between statistical models; M1 and M2, therefore other variable factors should be fixed. However, since the results derived from the *edgeR* can be uniquely observed, we additionally compared M1 and M2 in *DESeq2* for identifying differences between *edgeR* and *DESeq2*. As a result, high correlations (0.66 to 0.85) were observed between these two statistical tools (Additional file 1: Figure S14). In addition, larger numbers of DEGs were detected when using *DESeq2* (Additional file 1: Figure S15). Third, M2 has stronger statistical power than M1, which was observed in *edgeR* and *DESeq2* (Additional file 1: Figure S16). Finally, high tissue specificity was detected in *DESeq2* (Additional file 1: Figure S17). From these results, we conclude that although patterns of the test results from the *edgeR* and *DESeq2* are very similar, a larger number of DEGs and higher strong tissue specificity can be observed when using *DESeq2*.

In summary, although M2 was able to detect DEGs in multiple factorial RNA-seq data, M1 appears to be more suitable for the detection of sexually dimorphic genes with considering several tissue than M2 in terms of stability. As integrated model based approaches become more developed, those which consider heterogeneity variance and post-hoc analysis may provide a suitable way to detect DEGs in multi-factors designed RNA-seq data.

### Comparing detected sexually dimorphic genes between cattle and rat species

Upon performing technical verification, although gene expression patterns were shown to be different between cattle and rat, a large number of sexually dimorphic genes were commonly validated (Table 2; see also Fig. 5). Of 40 randomly selected candidate genes, 25 were commonly identified in both species including *CUX2, DDX3Y, USP9Y, ZNF280B*, and etc. In addition, 8 candidate genes, *APOD, CYP7A1, GABBR1, GNAL, SLC17A3, STXBP5L, TBL1X, and CATSPER2*, were technically validated in bovine qRT-PCR experiment, but those genes were not significant in rat species. In results of qRT-PCR analysis, 7 candidate genes were not significantly detected including *ADM, AMPD1, HOXD4, RGS9, SLC6A15, UCP3, and ME2*. Of these genes, only *ME2* was not significantly detected in either species; the others were significantly detected in only rat species. *CUX2* is well-known as a female-biased gene in mice [43]. As shown in Additional file 1: Figure S18, *CUX2* was shown to be female-biased gene rat liver tissue, but was identified as male-biased in cattle. *ZNF280B* and *RAB3C* showed diametrical patterns between the two species. These genes may serve as strong candidate markers for determination of sexual dimorphism in specific species. Of particular interest is *USP9Y*, which is a representative Y-linked gene. *USP9Y* has been known to show extremely low expression level in the brain of mice [44, 45], which is supported by our results (Fig. 5; see also Additional file 1: Figure S18). Contrastively, we observed that *USP9Y* was highly expressed in bovine whole tissues. Statistical qRT-PCR results showed that although *USP9Y* was not significantly detected in liver, muscle, and pituitary-gland tissues, it was significant in rat fat tissue (Table 2). For this reason, although *USP9Y* appears to not be significant in most rat tissues, the gene may be related to rodent sexual dimorphism. On the other hand, we observed high bovine expression levels of this gene, which indicate that *USP9Y* is strong candidate gene for identification of bovine sexually dimorphism.

Strong positive correlations among the tissues within the experiment were observed (Fig. 5-(d)) (0.79 to 0.9 for bovine RNA-seq, 0.43 to 0.89 for bovine qRT-PCR, and 0.18 to 0.58 for rat qRT-PCR). Of 40 DEGs randomly selected for qRT-PCR based technical validation, 33 sexually dimorphic genes were significantly detected in M2. M2 was able detect generally different genes across tissues (Additional file 1: Figure S6; see also Additional file 1: Figure S7). Strong positive correlations among tissues may be explained by specific features of M2. In order to examine this phenomenon, qRT-PCR and RNA-seq results were compared (Table 3). Of 33 significantly reported genes in RNA-seq, 21 were significantly identified using qRT-PCR. This result reveals that M2 is an attractive solution for detection of sexually dimorphic genes in multi-factorial designed RNA-seq data given its high reproducibility.

Finally, only 40 sexually dimorphic genes were used in order to compare the two species. While the need for cross-species transcriptome analysis has increased as interest in transcriptional evolution peaks, only a few studies have attempted to organize gene expression comparison among species [46]. Several factors make cross-species comparison of gene expression data difficult: (1) RNA sampling from the homologue across the species; (2) Construction of the orthologous gene-set across the species; (3) Normalization of the gene expression for different length gene; and (4) Compounding effects between species and other biases. Due to these reasons, most transcriptional cross-species studies have been performed at using meta-analysis based approaches rather than mega-analysis based ones [47–49]. Decisively, samples from multiple tissues should be matched in each meta-data set and considered in the gene expression study for sexually dimorphism given the highly tissue-specific pattern observed in our result. We performed qRT-PCR experiments on cattle and rat species to meet the minimum requirement for meta-study among multiple species. While our results do not compare a large number of sexually dimorphic genes, it may be beneficial in future studies to compare gene expression between species on a larger scale. As far as we know, this is first attempt to construct comparative heatmaps across species and multiple platforms (RNA-seq and microarray). Although these heatmaps provide relative gene expression patterns among three results, absolute intensities of color would be not important in cross-species comparison as most researchers would focus on detecting opposite tendencies among the species as was observed with *CUX2* and *USP9Y*. However, one limitation of qRT-PCR platform is that gene expression of genes can often be undetermined; some genes, including *UCP* in fat tissue and *SLC6A15* in liver and muscle tissue, showed strong blue intensity (Fig. 5-(a, c)). Development of methodology may be able to resolve this issue for future studies on RNA-seq based cross-species data.

## Conclusions

In order to investigate bovine sexually dimorphism, RNA-seq analysis was performed using two distinct statistical approaches. As a result, numerous sexually dimorphic genes and pathways were successfully identified across various tissues; expression showed strong tissue-specific patterns. For verification of the identified bovine sexually dimorphic genes, qRT-PCR experiments were performed in cattle and rat species, respectively. Results revealed that while sexually dimorphic genes are shared between these two mammal species, gene expression patterns vary across tissues. Results of our study have revealed that many biological processes might be involved in sexual dimorphism of metabolic tissues in cattle. Particularly, the expression patterns of sexually dimorphic genes in the pituitary gland indicated not only the effect of sexual dimorphism of the brain itself, but also of the central nervous system on peripheral tissues in determining differences between sexes. Finally, we concluded that two statistical approaches have their advantages and disadvantages in RNA-seq studies considering multiple tissues.

## Methods

### Animal handling and RNA-seq procedures

All animal procedures were approved by the National Institute of Animal Science Institutional Animal Use and Care Committee (NIASIAUCC), Republic of Korea, and performed in accordance with the animal experimental guidelines provided by NIASIAUCC. Samples were collected from Korean cattle raised in the Daekwanryung experimental branches of the National Institute of Animal Science (NIAS). 10 cattle were slaughtered at age of (>22 months) and carcass weight was 353 ± 36 kg after slaughter. Abdominal adipose tissue, liver, intact *longissimus dorsi* muscle, and pituitary gland tissue samples were immediately separated after slaughter. Tissue samples were stored at -80 °C, and total RNA was isolated from the four tissues using the TRIzol reagent (Invitrogen) based on the manufacturer instructions. Total RNA quality and quantity was verified using a NanoCrop1000 spectrophotometer (Thermo Scientific, Wilmington, DE, USA) and Bioanalyzer 2100 (Agilent technologies,Palo Alto CA, USA). The mRNA in total RNA was converted into a library of template molecules suitable for subsequent cluster generation using the reagents provided in the Illumina ® TruSeq™ RNA Sample Preparation Kit. In summary, mRNA was purified using poly-A selection, then chemically fragmented and converted into single-stranded cDNA using random hexamer priming. The second strand is then generated to create double-stranded cDNA that is ready for TruSeq library construction. The short ds-cDNA fragments were then connected with sequencing adapters, and suitable fragments were separated by agarose gel electrophoresis. Finally, truseq RNA libraries were built by PCR amplification, quantified using qPCR according to the qPCR Quantification Protocol Guide, qualified using the Agilent Technologies 2100 Bioanalyzer. (Agilent technologies,Palo Alto CA, USA). Based on the generated RNA libraries, paired-end sequencing (101 bp read-length and approximately 150 to 180 insert size) was performed using the HiSeq™ 2000 platform (Illumina,San Diego, USA). Next, to measure transcriptome levels with generated RNA-seq reads we performed the following widely used RNA-seq pipeline: (1) We employed Trimmomatic (v0.32) [15] with following option: PE -phred33 ILLUMINACLIP:TruSeq3-PE.fa:2:30:10 MINLEN:75 2 for making clean reads. (2) We mapped such clean reads into genome reference (BosTau7) from UCSC database using Bowtie2 (v2.2.2.0) [50], implemented within Tophat2 (v2.0.12) [51]. (3) For making the SAM file from the BAM file, we used a SAMtools (v0.1.18.0) [52]. (4) We used the HTseq package [53] to estimate the count of uniquely mapped reads for each of the 13,570 annotated genes in the *Bos taurus7* gene transfer format (. GTF) file. From this RNA-seq analysis pipeline, we obtained the transcriptome expression level of 13,570 genes from 40 samples.

### Statistical model for detecting sexual dimorphic genes in each tissue using GLM implemented in *edgeR*

In a previous study [6], diverse sexual dimorphic genes were detected by two group tests in four tissues using microarray data. Since gene expression data with enough replicates generally follows a normal distribution when using microarray analysis, a *t*-test (equivalent simple linear regression with only one explanatory two-group variable) can be applied to identify DEGs. However, approaches that consider count-type distribution such as Poisson and negative binomial (one solution for over-dispersion in Poisson assumption) is suitable for measuring gene expression from RNA-seq data given data characteristics [54]. Finally, GLM can be successfully used for analysis of RNA-seq data by considering gene expression as a negative binomial in *edgeR* in order to detect DEGs.

There are two approaches for identification of sexual dimorphic genes using RNA-seq data composed of two factors (sex and tissues). The simplest approach for analysis of this data is to perform a two group test between data from female and males in each tissue, separately, as has been performed in previous studies [6] using microarray data. To extend this method in RNA-seq analysis, we employed a GLM with only one explanatory variable (sex group variable) as shown in M1.

1 $$ \log \left(E(Y)\right) = \mu + Sex\kern0.24em \left[\mathbf{M}\mathbf{1}\right] $$

For identification of sexual dimorphic genes, we performed LRT using full model with sex term and reduced model without sex term in each tissue such as liver, fat, muscle, and pituitary-gland, respectively. We used an FDR < 0.05 significance cutoff [55] for multiple testing adjustment. Based on the list of genes found significant in each tissue, tissue specificities were calculated as the number of genes uniquely detected in a specific tissue over the total number of significantly detected genes in certain tissue [6].

### Integrated DE analysis using ANODEV for detection of sexual dimorphic genes in two-way factorial designed RNA-seq data

Recently, novel statistical methods optimized for complex RNA-seq experimental designs have been developed [13, 56]. In a GLM, ANODEV can be applied in the place of ANOVA for analysis of RNA-seq data displaying a count-based distribution. By including an additional factor following the linear predictor, the GLM is easily extended

2 $$ \log \left({\theta}_{ijk}\right) = {\mu}_j + {\tau}_{ij} + {\beta}_{jk} $$

where, *i* is treatment, *j* is gene, and *k* is individual. As shown in log-link function and related linear predictor (2), only *β*_*jk*_ is different between M1 (including only one group explanatory variable) and M2. By including *β*_*jk*_ with several covariates such as tissue, the GLM can easily be extended into an ANOVA type model. In this paper, our model simultaneously considered two factors, sex and tissue. This model can more simply be laid out by expressing the linear predictor as following an ANOVA type model as follows:

3 $$ \log \left(E(Y)\right) = \mu + Sex + Tissue\kern0.49em \left[\mathbf{M}\mathbf{2}\right] $$

As shown in M2, we simultaneously considered the effect of two factors on the model. By considering two factors, the design matrix was composed of 40 (Sample size) * 5 (Intercept + level of sex -1 + level of tissue - 1). Considering our goal of identifying sex differences, our term of interest in this model is sex. In order to observe DEGs between males and females, we performed a LRT on each gene related to sex (13,148 genes remained after filtering non-expressed genes). From the statistical test, significant (FDR-Adjusted *P*-Value < 0.05) genes were detected to adjust for multiple testing errors [55].

### Gene-set and chromosomal enrichment analyses using DAVID and a fisher’s exact test, respectively, for biological interpretation

Several previous studies have found that sexual dimorphic genes are enriched in the sex chromosome, particularly the X-chromosome [6, 57, 58]. We performed a chromosomal enrichment test in order to investigate this phenomenon in cattle. We employed a fisher’s exact test with a 2x2 contingency table, composed of two factors: whether the gene was included in a specific chromosome (O / X), and whether the gene was a DEG or not (O/X). 31 different contingency tables were produced from each chromosome. We performed a fisher’s exact test using the table with the alternative hypothesis that one odds ratio was larger than the other. Additionally, gene-set enrichment analysis was performed using DAVID with default options [59] after detection of DEGs using M1 and M2, respectively. “*Bos taurus*” was set as the background reference. Several gene-set databases were cross-referenced within DAVID including KEGG and Gene Ontology. We performed statistical tests using resulting information and the DEG list in order to calculate the significance of each gene-set related to sexual dimorphism.

### qRT-PCR experiments on Korean cattle and Sprague Dawley rat for technical validation of sexual dimorphic genes

- Korean cattle handling and tissue extraction
  Four male and female Korean cattle were provided by the Daekwanryung branches of the National Institute of Animal Science (NIAS), Republic of Korea. Cattle were slaughtered at approximately 22 months old and average carcass weight was recorded as 366.17 ± 11.22 kg and 341.50 ± 18.95 kg for bulls and cows, respectively. 4 different kinds of tissue samples were collected: abdominal adipose, intact *longissimus dorsi* muscle, liver, and pituitary gland. Samples were immediately separated after slaughter and stored at -80 °C. All animal experimental procedures were approved by NIASIAUCC and managed in accordance with the animal experimental guidelines provided by NIASIAUCC.
- Sprague Dawley rat handling and tissue extraction
  Sprague Dawley (SD) male and female outbred rats (8 weeks old, 180–240 g, *n* = 5, respectively) were obtained from Koatech, Inc. (Gyunggi-Do, Korea) and maintained in a temperature (22 ± 1 °C) and humidity (45–65 %) controlled room on a 12:12 light:dark cycle. They were allowed access to normal food and water *ad libitum* for recovery according to specific pathogen-free (SPF) conditions. All animal experimental procedures were approved by NIASIAUCC and managed in accordance with the animal experimental guidelines provided by NIASIAUCC. After 6 h of fasting, samples were collected from 4 different tissues: abdominal adipose tissue, skeletal muscle, liver, and pituitary gland from each of the 10 rats. All surgical instruments were pre-sterilized by steam sterilization. After body weight was measured and recorded, rats were anesthetized with CO_2_ and placed on an operating table. Tissues were quickly removed after sacrifice, washed with PBS, and then chilled in liquid nitrogen. For determination of mRNA levels, samples were stored at -80 °C. All tissues were collected from male and female SD rats.
- RNA extraction and real-time polymerase chain reaction analysis
  All tissues were collected from male and female Korean cattle and SD rats. Total RNA was isolated from 4 different tissues using the TRIzol reagent (Invitrogen Carlsbad, CA, USA) according to manufacturer instructions. The extracted total RNA was stored at -80 °C and then assessed through real-time PCR and RNA-sequencing. Briefly, total RNA samples were quantified by absorbance at 260/280 nm ratio. Total RNA (≤ 1 μg) was transcribed into cDNA using QuantiTect Reverse Transcription Kit (Qiagen, Valencia, CA, USA) and real-time PCR was performed using SYBR Green PCR Master Mix (Qiagen). Glyceraldehyde-3-phosphate dehydrogenase (*GAPDH*), β-actin (*ACTB*), ribosomal protein S9 (RPS9), and ribosomal protein, large, P0 (*RPLP0*) were used as endogenous control genes for the measurement of fold change. Primers are listed in Additional file 1: Table S19 and Table S20 for cattle and rat, respectively.
- Quantile normalization between RNA-seq and qRT-PCR in order to visualize a relative heatmap with adjusting ranges in two platforms
  To draw a comparative heatmap using RNA-seq and qRT-PCR, it is important to normalize gene expression values since scale and range of calculated gene expressions from qRT-PCR and RNA-seq vary from each other. For example, ΔCt values from the qRT-PCR ranged in − ∞ to ∞, and smaller value means high-level gene expression. On the other hand, gene expression of RNA-seq (log2 transformed TMM values by *edgeR*) is non-negative values and larger value means high-level gene expression. We corrected the range of ΔCt values as non-negative scale by adding an absolute minimum ΔCt value after multiplying ΔCt by -1. After that, we performed quantile normalization to adjust scale among the two platforms.

### Availability of supporting data

The data sets supporting the results of this article is available in the GEO database, GSE65125 in http://www.ncbi.nlm.nih.gov/geo/query/acc.cgi?acc=GSE65125.

## Additional files

Additional file 1:
**Additional figures and tables.** (PDF 3440 kb) Additional file 2:
**Results of the Statistical tests.** (XLSX 2242 kb)

## Abbreviations

ANODEV: Analysis of deviance
qRT-PCR: Quantitative real time PCR
CNS: Central nervous system
GH: Growth hormone
LH: Luteinizing hormone
FSH: Follicle stimulating hormone
DEGs: Differentially expressed genes
ANOVA: Analysis of variance
GTF: General Transfer Format
XCI: X-chromosome inactivation
GWAS: Genome-wide association studies
SNPs: Single nucleotide polymorphisms
LRT: Likelihood ratio test
GLM: Generalized linear model
WLSE: Weighted least squares
GEE: Estimating equation
NIAS: National Institute of Animal Science
