# Supplementary material for: Comprehensive identification of sexually dimorphic genes in diverse cattle tissues using RNA-seq
Source: BMC Genomics. 2016 Jan 27;17:81. doi: 10.1186/s12864-016-2400-4 (PMC4728830; doi:10.1186/s12864-016-2400-4)
Supplement: Additional file 1: — Additional figures and tables. (PDF 3440 kb) [file 12864_2016_2400_MOESM1_ESM.pdf]

Comprehensive identification of sexually dimorphic genes in diverse cattle tissues using RNA-seq

Supplementary Tables

Table S1. Clean reads rate and mapping rate 4

Table S2. Number of annotated genes in each chromosome (autosomes and sex-chromosomes) 6

Table S3. Enrichment analysis for male-biasness based on the Fisher's-exact test 7

Table S4. Number of significant detected DEGs in each chromosome (FDR adjusted P-value < 0.05) 8

Table S5. Top-20 most significant genes from the M2 model out of a total of 655. 9

Table S6. Proportion of overlapped genes between M1 (each-tissue model) and M2 (integrated model) 10

Table S7. Significant biological terms from DAVID enrichment test with DEGs from the M2 (FDR < 0.05) based on several biological databases such as gene ontology and KEGG. 11

Table S8. Set of 14 detected clusters of biological terms derived from DAVID annotation clustering. 13

Table S9. Primer sequences used in real-time PCR analysis of cattle tissues 17

Table S10. Primer sequences used in real-time PCR analysis of rat tissues 19

Table S11. The significant gene set from DAVID enrichment test with DEGs from fat tissue (FDR < 0.2) and several gene-set databases such as gene ontology and KEGG. 21

Table S12. The significant gene set from DAVID enrichment test with DEGs from liver tissue (FDR < 0.2) and several gene-set databases such as gene ontology and KEGG. 22

Table S13. The significant gene set from DAVID enrichment test with DEGs from muscle tissue (FDR < 0.2) and several gene-set databases such as gene ontology and KEGG. 23

Table S14. The significant gene set from DAVID enrichment test with DEGs from pituitary-gland tissue (FDR < 0.2) and several gene-set databases such as gene ontology and KEGG. 27

Supplementary Figures

Figure S1. Exploration of gender biases and chromosomal effects on tissues 30

Figure S2. Manhattan plot for allocating sexual dimorphic genes from located chromosomal position with y-axis is a –log10 transformed FDR adjusting P-values 31

Figure S3. Overlapped Manhattan plot across the all tissues for allocating sexual dimorphic genes from located chromosomal position with y-axis is a log2 fold changes between female and male. Total 13,148 genes were visualized in this plot. 32

Figure S4. Separated Manhattan plots for significantly observed genes in M1 33

Figure S5. Venn-diagram for M1 (each-tissue model) and M2 (integrated model) 34

Figure S6. Box plots for comparison between M1 and M2. (A) Representative significant genes in M1 (tissue separately model). (B) Representative significant genes in M2 (Integrated model). Y-axis represent normalized values by TMM normalization using edgeR and X-axis represent gender. Four colored lines were drew in each tissues, which is connected between average of female and male expression values, respectively. 35

Figure S7. Line plots for comparison between M1 and M2. (A) Representative significant genes in M1 (tissue separately model). (B) Representative significant genes in M2 (Integrated model). Y-axis represent normalized values by TMM normalization using edgeR and X-axis represent gender. Four colored lines were drew in each tissues, which is connected between average of female and male expression values, respectively. 36

Figure S8. Manhattan plots. (A) FDR adjusted P-value of the M1 method for each sampled tissue and chromosomal location. (B) FDR adjusted P-value of the M2 method. Red marked genes are significant in M2 under FDR < 0.05. 37

Figure S9. Visualizing densities of the FDR adjusted p-values from M1 for 655 detected DEGs from M2. The red-vertical line represents 5% significance level. 38

Figure S10. Hierarchical clustering analysis of 40 sexual dimorphic genes for qRT-PCR validation of RNA-seq results. Three trees were visualized with optimal number of clusters based on the Silhouette score. The colors represent estimated groups derived from hierarchical clustering and its optimal number of clusters, respectively. (A) qRT-PCR for cattle species with 32 samples (k=7). (B) RNA-seq result for cattle species (k=4). (C) qRT-PCR for rat species with 40 samples (k=4). (D) Silhouette score plot for detecting optimal number of clusters. 39

Figure S11. Gene expression patterns for annotating 8 Y-linked genes based on bosTau7 genome. 40

Figure S12. Detected three male-over-expressed genes (FDR adjusted P-value < 0.1) 41

Figure S13. Box-plot describing gene expression of XIST 42

Figure S14. The correlation plot for comparison between two statistical analysis tools such as edgeR and DESeq2. Spearman correlation coefficients were employed with each P-value derived from different hypothesis tests. Higher correlation coefficients (0.66 to 0.85) were observed in same hypothesis tests

across the edgeR and DESeq2. 43

Figure S15. Venn diagram of DEGs list (FDR adjusted P-value < 0.05) for comparison between two statistical analysis tools such as edgeR and DESeq2. In the DESeq2, relatively large number of DEGs were significantly detected compared to the edgeR. 44

Figure S16. Venn diagram of DEGs list (FDR adjusted P-value < 0.05) for comparison between two statistical analysis tools such as edgeR and DESeq2. The lager number of detected genes in M1 than M2, which were observed in edgeR and DESeq2. 45

Figure S17. Tissue specificity of the detected DEGs using edgeR and DESeq2, respectively. Number of detected genes and tissue specific genes by FDR cutoff and their tissue specificity calculation. While edgeR and DESeq2 generate almost same result (Relatively Higher tissue specificity of the Muscle and pituitary gland and relatively lower tissue specificity of the liver and fat tissue) 46

Figure S18. Comparative heatmap between bovine and rat qRT-PCR results. The intensities represent FM:M ratio (Female gene expression / Male gene expression). 47

Table S1. Clean reads rate and mapping rate

Sample index	# of surviving reads	rate of surviving reads	Mapping rate
**Sample 1**	26179974	99.62%	87.80%
**Sample 2**	25503691	99.50%	87.10%
**Sample 3**	19064029	89.72%	85.20%
**Sample 4**	20322303	99.58%	84.30%
**Sample 5**	20076920	99.46%	85.30%
**Sample 6**	21938115	99.21%	75.60%
**Sample 7**	25050800	99.20%	82.30%
**Sample 8**	22777313	99.61%	83.80%
**Sample 9**	22708528	98.80%	88.20%
**Sample 10**	19883540	99.56%	85.60%
**Sample 11**	20330861	99.00%	87.30%
**Sample 12**	21300243	99.29%	84.80%
**Sample 13**	17647513	99.56%	86.20%
**Sample 14**	23809821	99.11%	81.30%
**Sample 15**	23366099	99.55%	82.90%
**Sample 16**	17000674	99.69%	83.50%
**Sample 17**	18827861	98.82%	87.00%
**Sample 18**	18400226	93.04%	71.00%
**Sample 19**	20686365	99.22%	84.50%
**Sample 20**	19809624	99.29%	82.80%
**Sample 21**	17093784	99.62%	85.50%
**Sample 22**	15789538	99.67%	84.20%
**Sample 23**	17106191	99.61%	85.60%
**Sample 24**	16552140	99.50%	83.20%
**Sample 25**	13505439	93.99%	60.20%
**Sample 26**	26228360	97.65%	71.50%
**Sample 27**	28451747	97.08%	70.70%
**Sample 28**	28189426	98.12%	71.60%
**Sample 29**	16430150	99.25%	85.70%
**Sample 30**	16241661	99.78%	82.80%
**Sample 31**	22247985	99.40%	76.50%
**Sample 32**	18015690	99.53%	81.40%
**Sample 33**	32493117	99.72%	84.60%
**Sample 34**	16722272	99.63%	82.90%
**Sample 35**	15578083	99.50%	84.80%
**Sample 36**	32141655	99.56%	81.40%
**Sample 37**	25664836	99.34%	85.30%

**Sample 38**	23968355	99.15%	76.50%
**Sample 39**	19579272	99.63%	83.10%
**Sample 40**	20413006	99.34%	82.30%
**AVERAGE**	21177430.18	98.75%	81.91%

Table S2. Number of annotated genes in each chromosome (autosomes and sex-chromosomes)

Chr.	# of genes	Chr.	# of genes	Chr.	# of genes
chr1	535	chr12	221	chr23	400
chr2	593	chr13	480	chr24	168
chr3	819	chr14	263	chr25	476
chr4	435	chr15	443	chr26	241
chr5	774	chr16	363	chr27	141
chr6	378	chr17	364	chr28	183
chr7	733	chr18	700	chr29	364
chr8	431	chr19	811	chrUn	538
chr9	288	chr20	205	chrX	350
chr10	545	chr21	354	chrY	10
chr11	584	chr22	380	Total : 13,570	

Table S3. Enrichment analysis for male-biasness based on the Fisher's-exact test

Tissues	Number of Male-biased genes	Number of Female-biased genes	Criteria for DEG	Enrichment analysis for Male-biased
**Liver**	8	16	FDR < 0.05	0.803
**Fat**	6	8	FDR < 0.05	0.491
**Muscle**	23	63	FDR < 0.05	0.999
**Pituitary-gland**	34	23	FDR < 0.05	0.0001*
**Liver**	13	32	FDR < 0.1	0.727
**Fat**	7	11	FDR < 0.1	0.3327
**Muscle**	34	129	FDR < 0.1	1
**Pituitary-gland**	52	56	FDR < 0.1	9.61E-06*
**Liver**	34	95	FDR < 0.2	0.474
**Fat**	8	20	FDR < 0.2	0.437
**Muscle**	70	348	FDR < 0.2	1
**Pituitary-gland**	94	130	FDR < 0.2	1.83E-10*

(*) Significant result with P-value < 0.05

The Fisher's exact test was employed for significance test of the male-biasness in each tissue. The hypothesis test was performed using 2x2 contingency tables composed of two factors such as Gender-biasness (Male-biased gene / Female-biased gene) and Tissue (Targeted tissue / The others). The alternative hypothesis is the odds ratio is greater than 1, which means male-biased genes of the specific tissue are significantly enriched compared to other tissues.

Table S4. Number of significant detected DEGs in each chromosome (FDR adjusted P-value < 0.05)

Chr.	**Liver**	**Fat**	**Muscle**	**Pit.**	Chr.	**Liver**	**Fat**	**Muscle**	**Pit.**
**Chr. 1**	0	0	4	1	Chr. 17	2	0	2	2
**Chr. 2**	1	0	7	3	Chr. 18	0	0	4	2
**Chr. 3**	2	1	3	1	Chr. 19	3	1	8	3
**Chr. 4**	2	0	3	2	Chr. 20	0	0	1	0
**Chr. 5**	0	1	5	3	Chr. 21	0	0	0	3
**Chr. 6**	0	2	2	3	Chr. 22	0	0	1	0
**Chr. 7**	0	0	3	0	Chr. 23	0	0	1	2
**Chr. 8**	2	0	1	3	Chr. 24	2	0	2	2
**Chr. 9**	0	0	5	2	Chr. 25	0	0	1	2
**Chr. 10**	1	0	3	0	Chr. 26	0	1	1	2
**Chr. 11**	0	0	4	3	Chr. 27	0	0	1	0
**Chr. 12**	1	0	1	0	Chr. 28	0	0	2	1
**Chr. 13**	0	0	0	2	Chr. 29	1	0	0	1
**Chr. 14**	1	0	0	2	Chr. X	0	1	5	3
**Chr. 15**	2	1	3	1	Chr. Y	3	3	3	3
**Chr. 16**	0	1	4	2	Chr. Un	1	2	6	3

Table S5. Top-20 most significant genes from the M2 model out of a total of 655.

Gene_Symbol	Chromosome	FDR adjusting P-value
USP9Y	chrY	5.47E-72
DDX3Y	chrY	6.56E-55
ZFY	chrY	8.79E-55
XIST	chrX	1.37E-17
LOC780876	chr6	3.71E-12
LYSB	chr5	5.71E-06
GABBR1	chr23	4.30E-05
CYP7A1	chr14	6.19E-05
KDM6A	chrX	9.01E-05
NR2E1	chr9	0.000257
NRBP2	chr14	0.000372
COL27A1	chr8	0.000424
DOCK6	chr7	0.000466
MMP12	chr15	0.000595
WDR19	chr6	0.000595
VSIG2	chr29	0.000623
SRSF2	chr19	0.000711
RGS9	chr19	0.000765
GNAL	chr24	0.000968
AMPD1	chr3	0.001046

Table S6. Proportion of overlapped genes between M1 (each-tissue model) and M2 (integrated model)

FDR cutoff	Liver	Fat	Muscle	Pituitary-gland
< 0.05	0.583	0.571	0.453	0.403
< 0.1	0.577	0.888	0.533	0.481
< 0.2	0.604	0.928	0.622	0.517

Table S7. Significant biological terms from DAVID enrichment test with DEGs from the M2 (FDR < 0.05) based on several biological databases such as gene ontology and KEGG.

Category	Term	P-Value	Count
KEGG_PATHWAY	Calcium signaling pathway	3.70E-03	13
KEGG_PATHWAY	Fructose and mannose metabolism	1.20E-02	5
KEGG_PATHWAY	Autoimmune thyroid disease	3.90E-02	5
KEGG_PATHWAY	Circadian rhythm	4.90E-02	3
KEGG_PATHWAY	Glycosphingolipid biosynthesis	6.50E-02	3
KEGG_PATHWAY	Primary immunodeficiency	7.10E-02	4
KEGG_PATHWAY	Taste transduction	7.60E-02	4
KEGG_PATHWAY	Hematopoietic cell lineage	8.50E-02	6
KEGG_PATHWAY	T cell receptor signaling pathway	9.60E-02	7
KEGG_PATHWAY	Natural killer cell mediated cytotoxicity	9.60E-02	7
KEGG_PATHWAY	Steroid hormone biosynthesis	9.90E-02	4
GOTERM_BP_FAT	regulation of RNA metabolic process	2.90E-04	50
GOTERM_BP_FAT	transcription	4.00E-04	33
GOTERM_BP_FAT	rhythmic process	6.90E-04	7
GOTERM_BP_FAT	regulation of transcription, DNA-dependent	1.20E-03	47
GOTERM_BP_FAT	regulation of transcription	2.40E-03	59
GOTERM_BP_FAT	leukocyte activation	3.80E-03	11
GOTERM_BP_FAT	eye photoreceptor cell development	5.50E-03	4
GOTERM_BP_FAT	eye photoreceptor cell differentiation	7.20E-03	4
GOTERM_BP_FAT	activation of adenylate cyclase activity	1.10E-02	4
GOTERM_BP_FAT	photoreceptor cell development	1.10E-02	4
GOTERM_BP_FAT	cell activation	1.10E-02	11
GOTERM_BP_FAT	lymphocyte activation	1.20E-02	9
GOTERM_BP_FAT	positive regulation of adenylate cyclase activity	1.40E-02	4
GOTERM_BP_FAT	photoreceptor cell differentiation	1.40E-02	4
GOTERM_BP_FAT	positive regulation of cyclase activity	1.60E-02	4
GOTERM_BP_FAT	positive regulation of lyase activity	2.30E-02	4
GOTERM_BP_FAT	modification-dependent macromolecule catabolic process	2.50E-02	15
GOTERM_BP_FAT	modification-dependent protein catabolic process	2.50E-02	15
GOTERM_BP_FAT	eye morphogenesis	2.90E-02	5
GOTERM_BP_FAT	vesicle-mediated transport	3.00E-02	16
GOTERM_BP_FAT	hemopoiesis	3.30E-02	9
GOTERM_BP_FAT	regulation of adenylate cyclase activity	3.40E-02	4
GOTERM_BP_FAT	ovulation cycle process	3.40E-02	4
GOTERM_BP_FAT	female gonad development	3.40E-02	4
GOTERM_BP_FAT	ovulation cycle	3.40E-02	4

GOTERM_BP_FAT	intracellular signaling cascade	3.80E-02	26
GOTERM_BP_FAT	female sex differentiation	3.90E-02	4
GOTERM_BP_FAT	development of primary female sexual characteristics	3.90E-02	4
GOTERM_BP_FAT	regulation of cellular response to stress	4.10E-02	5
GOTERM_BP_FAT	gonad development	4.10E-02	5
GOTERM_BP_FAT	secretion by cell	4.40E-02	8
GOTERM_BP_FAT	regulation of interleukin-2 biosynthetic process	4.50E-02	3
GOTERM_BP_FAT	proteolysis involved in cellular protein catabolic process	4.70E-02	15
GOTERM_BP_FAT	regulation of cyclase activity	4.90E-02	4
GOTERM_BP_FAT	cellular protein catabolic process	4.90E-02	15
GOTERM_BP_FAT	calcium ion-dependent exocytosis	5.30E-02	3
GOTERM_BP_FAT	regulation of cAMP biosynthetic process	5.40E-02	4
GOTERM_BP_FAT	development of primary sexual characteristics	5.60E-02	5
GOTERM_BP_FAT	neuron development	5.80E-02	8
GOTERM_BP_FAT	hemopoietic or lymphoid organ development	5.80E-02	9
GOTERM_BP_FAT	regulation of lyase activity	5.90E-02	4
GOTERM_BP_FAT	reproductive structure development	6.00E-02	5
GOTERM_BP_FAT	eye development	6.30E-02	6
GOTERM_BP_FAT	regulation of cAMP metabolic process	7.10E-02	4
GOTERM_BP_FAT	regulation of adenylate cyclase activity involved in G-protein signaling	7.20E-02	3
GOTERM_BP_FAT	cytolysis	7.20E-02	3
GOTERM_BP_FAT	positive regulation of adenylate cyclase activity by G-protein signaling pathway	7.20E-02	3
GOTERM_BP_FAT	activation of adenylate cyclase activity by G-protein signaling pathway	7.20E-02	3
GOTERM_BP_FAT	adult behavior	7.40E-02	5
GOTERM_BP_FAT	cellular macromolecule catabolic process	7.80E-02	16
GOTERM_BP_FAT	second-messenger-mediated signaling	7.90E-02	5
GOTERM_BP_FAT	protein catabolic process	8.10E-02	15
GOTERM_BP_FAT	lymphocyte differentiation	8.30E-02	5
GOTERM_BP_FAT	regulation of cyclic nucleotide biosynthetic process	8.40E-02	4
GOTERM_BP_FAT	regulation of nucleotide biosynthetic process	8.40E-02	4
GOTERM_BP_FAT	immune system development	8.40E-02	9

Table S8. Set of 14 detected clusters of biological terms derived from DAVID annotation clustering.

Enrichment Score: 2.91		P-value
GOTERM_BP_FAT	transcription	4.00E-04
**Enrichment Score: 2.48**		**P-value**
GOTERM_BP_FAT	regulation of RNA metabolic process	2.90E-04
GOTERM_BP_FAT	transcription	4.00E-04
GOTERM_BP_FAT	regulation of transcription, DNA-dependent	1.20E-03
GOTERM_MF_FAT	transcription factor activity	1.60E-03
GOTERM_BP_FAT	regulation of transcription	2.40E-03
GOTERM_MF_FAT	DNA binding	2.20E-02
GOTERM_MF_FAT	transcription regulator activity	2.70E-02
GOTERM_MF_FAT	sequence-specific DNA binding	7.20E-02
**Enrichment Score: 2.32**		**P-value**
GOTERM_MF_FAT	cation binding	1.30E-04
GOTERM_MF_FAT	ion binding	1.90E-04
GOTERM_MF_FAT	metal ion binding	3.20E-04
GOTERM_MF_FAT	transition metal ion binding	4.60E-03
GOTERM_MF_FAT	zinc ion binding	1.80E-02
**Enrichment Score: 2.01**		**P-value**
GOTERM_MF_FAT	nucleotide binding	2.70E-04
GOTERM_MF_FAT	purine nucleotide binding	3.30E-03
GOTERM_MF_FAT	ribonucleotide binding	4.60E-03
GOTERM_MF_FAT	purine ribonucleotide binding	4.60E-03
GOTERM_MF_FAT	purine nucleoside binding	6.50E-03
GOTERM_MF_FAT	ATP binding	6.70E-03
GOTERM_MF_FAT	nucleoside binding	7.20E-03
GOTERM_MF_FAT	adenyl ribonucleotide binding	7.80E-03
GOTERM_MF_FAT	adenyl nucleotide binding	8.60E-03
GOTERM_MF_FAT	protein kinase activity	1.90E-01
GOTERM_BP_FAT	phosphorylation	1.90E-01
**Enrichment Score: 1.92**		**P-value**
GOTERM_BP_FAT	modification-dependent macromolecule catabolic process	2.50E-02
GOTERM_BP_FAT	modification-dependent protein catabolic process	2.50E-02
GOTERM_BP_FAT	proteolysis involved in cellular protein catabolic process	4.70E-02
GOTERM_BP_FAT	cellular protein catabolic process	4.90E-02
GOTERM_BP_FAT	cellular macromolecule catabolic process	7.80E-02
GOTERM_BP_FAT	protein catabolic process	8.10E-02
GOTERM_BP_FAT	macromolecule catabolic process	1.60E-01
GOTERM_BP_FAT	proteolysis	3.80E-01

**Enrichment Score: 1.32**		**P-value**
GOTERM_BP_FAT	RNA processing	9.00E-01
**Enrichment Score: 1.21**		**P-value**
GOTERM_CC_FAT	external side of plasma membrane	4.90E-02
GOTERM_CC_FAT	cell surface	5.60E-02
KEGG_PATHWAY	Hematopoietic cell lineage	8.50E-02
**Enrichment Score: 1.19**		**P-value**
GOTERM_BP_FAT	rhythmic process	6.90E-04
GOTERM_BP_FAT	ovulation cycle process	3.40E-02
GOTERM_BP_FAT	ovulation cycle	3.40E-02
GOTERM_BP_FAT	female gonad development	3.40E-02
GOTERM_BP_FAT	development of primary female sexual characteristics	3.90E-02
GOTERM_BP_FAT	female sex differentiation	3.90E-02
GOTERM_BP_FAT	gonad development	4.10E-02
KEGG_PATHWAY	Circadian rhythm	4.90E-02
GOTERM_BP_FAT	development of primary sexual characteristics	5.60E-02
GOTERM_BP_FAT	reproductive structure development	6.00E-02
GOTERM_BP_FAT	sex differentiation	1.20E-01
GOTERM_BP_FAT	reproductive developmental process	2.40E-01
GOTERM_MF_FAT	copper ion binding	4.10E-01
GOTERM_BP_FAT	multicellular organism reproduction	5.80E-01
GOTERM_BP_FAT	reproductive process in a multicellular organism	5.80E-01
**Enrichment Score: 1.17**		**P-value**
GOTERM_BP_FAT	leukocyte activation	3.80E-03
GOTERM_BP_FAT	cell activation	1.10E-02
GOTERM_BP_FAT	lymphocyte activation	1.20E-02
GOTERM_BP_FAT	hemopoiesis	3.30E-02
GOTERM_BP_FAT	hemopoietic or lymphoid organ development	5.80E-02
GOTERM_BP_FAT	lymphocyte differentiation	8.30E-02
GOTERM_BP_FAT	immune system development	8.40E-02
GOTERM_BP_FAT	B cell activation	1.20E-01
GOTERM_BP_FAT	T cell differentiation in the thymus	1.50E-01
GOTERM_BP_FAT	T cell activation	1.50E-01
GOTERM_BP_FAT	leukocyte differentiation	1.60E-01
GOTERM_BP_FAT	T cell differentiation	3.30E-01
GOTERM_BP_FAT	homeostasis of number of cells	6.00E-01
**Enrichment Score: 1.13**		**P-value**
GOTERM_BP_FAT	activation of adenylate cyclase activity	1.10E-02
GOTERM_BP_FAT	positive regulation of adenylate cyclase activity	1.40E-02

GOTERM_BP_FAT	positive regulation of cyclase activity	1.60E-02
GOTERM_BP_FAT	positive regulation of lyase activity	2.30E-02
GOTERM_BP_FAT	regulation of adenylate cyclase activity	3.40E-02
GOTERM_BP_FAT	regulation of cyclase activity	4.90E-02
GOTERM_BP_FAT	regulation of cAMP biosynthetic process	5.40E-02
GOTERM_BP_FAT	regulation of lyase activity	5.90E-02
GOTERM_BP_FAT	regulation of cAMP metabolic process	7.10E-02
GOTERM_BP_FAT	positive regulation of adenylate cyclase activity by G-protein signaling pathway	7.20E-02
GOTERM_BP_FAT	activation of adenylate cyclase activity by G-protein signaling pathway	7.20E-02
GOTERM_BP_FAT	regulation of adenylate cyclase activity involved in G-protein signaling	7.20E-02
GOTERM_BP_FAT	second-messenger-mediated signaling	7.90E-02
GOTERM_BP_FAT	regulation of cyclic nucleotide biosynthetic process	8.40E-02
GOTERM_BP_FAT	regulation of nucleotide biosynthetic process	8.40E-02
GOTERM_BP_FAT	regulation of cyclic nucleotide metabolic process	1.10E-01
GOTERM_BP_FAT	regulation of nucleotide metabolic process	1.20E-01
GOTERM_BP_FAT	positive regulation of catalytic activity	1.40E-01
GOTERM_BP_FAT	G-protein signaling, coupled to cAMP nucleotide second messenger	1.60E-01
GOTERM_BP_FAT	G-protein signaling, coupled to cyclic nucleotide second messenger	1.60E-01
GOTERM_BP_FAT	cAMP-mediated signaling	1.80E-01
GOTERM_BP_FAT	cyclic-nucleotide-mediated signaling	1.90E-01
GOTERM_BP_FAT	positive regulation of molecular function	2.60E-01
GOTERM_BP_FAT	positive regulation of hydrolase activity	4.90E-01
**Enrichment Score: 1.12**		**P-value**
GOTERM_BP_FAT	secretion by cell	4.40E-02
GOTERM_BP_FAT	calcium ion-dependent exocytosis	5.30E-02
GOTERM_BP_FAT	secretion	1.20E-01
GOTERM_BP_FAT	exocytosis	1.20E-01
**Enrichment Score: 1.1**		**P-value**
GOTERM_MF_FAT	motor activity	1.10E-02
GOTERM_MF_FAT	microtubule motor activity	9.80E-02
GOTERM_BP_FAT	microtubule-based process	1.80E-01
GOTERM_BP_FAT	microtubule-based movement	2.10E-01
**Enrichment Score: 1.07**		**P-value**
GOTERM_BP_FAT	eye photoreceptor cell development	5.50E-03
GOTERM_BP_FAT	eye photoreceptor cell differentiation	7.20E-03
GOTERM_CC_FAT	cilium part	7.50E-03
GOTERM_BP_FAT	photoreceptor cell development	1.10E-02
GOTERM_BP_FAT	photoreceptor cell differentiation	1.40E-02
GOTERM_BP_FAT	eye morphogenesis	2.90E-02

GOTERM_BP_FAT	neuron development	5.80E-02
GOTERM_BP_FAT	eye development	6.30E-02
GOTERM_CC_FAT	nonmotile primary cilium	1.20E-01
GOTERM_BP_FAT	neuron differentiation	1.30E-01
GOTERM_CC_FAT	cell projection	1.60E-01
GOTERM_CC_FAT	cilium	2.00E-01
GOTERM_BP_FAT	sensory perception of light stimulus	2.10E-01
GOTERM_BP_FAT	visual perception	2.10E-01
GOTERM_BP_FAT	sensory organ development	2.30E-01
GOTERM_CC_FAT	cell projection part	2.50E-01
GOTERM_BP_FAT	sensory perception of chemical stimulus	3.00E-01
GOTERM_BP_FAT	sensory perception	3.20E-01
GOTERM_BP_FAT	cognition	3.60E-01
GOTERM_BP_FAT	neurological system process	4.00E-01
GOTERM_BP_FAT	camera-type eye development	5.30E-01
**Enrichment Score: 1.04**		**P-value**
KEGG_PATHWAY	Fructose and mannose metabolism	1.20E-02
GOTERM_MF_FAT	phosphofructokinase activity	1.60E-02
GOTERM_MF_FAT	carbohydrate kinase activity	1.00E-01
GOTERM_BP_FAT	hexose metabolic process	5.50E-01
GOTERM_BP_FAT	monosaccharide metabolic process	6.30E-01

Table S9. Primer sequences used in real-time PCR analysis of cattle tissues

Gene name	Symbol	GenBank ID	5' → 3'	Sequences	Product size, bp
Adrenomedullin	ADM	BC123826	Forward	TTTCACGGACAAGGACAAGG	118
			Reverse	CTGTAATAGAGTCCGACCCAAG	
Adenosine monophosphate deaminase 1	AMPD1	NM_001100349	Forward	TCTATCCAGTCTTTACCCCTCC	105
			Reverse	CCACACCGTCCTTCATTTTG	
Apolipoprotein D	APOD	NM_001076301	Forward	GCCACCGACTATGAGAACTAC	124
			Reverse	GATAGGTCACTGTTTCTGGAGG	
Rho guanine nucleotide exchange factor (GEF) 19	ARHGEF19	NM_001206270	Forward	TTCCTCATCCTGCCGTTTC	95
			Reverse	ATGTCTTCATCTTCAGAGCCC	
Cation channel, sperm associated 2	CATSPER2	NM_001192477	Forward	AGAACGGATTACACATGCCC	109
			Reverse	GAAGATAGGACGCTCAAGGAC	
Glycoprotein hormones, alpha polypeptide	CGA	NM_173901	Forward	TCCAATCTATCAGTGCATGGG	136
			Reverse	GGCCTTGGTAAATGCTTTGG	
Collagen, type XXVII, alpha 1	COL27A1	NM_001206680	Forward	CGGGTCCAGATGAATTTCCTG	73
			Reverse	TGTTAAGGCAGTGGATGGTG	
Cut-like homeobox 2	CUX2	NM_001192597	Forward	CAGTATCACCCCAAGAATCCG	97
			Reverse	CTTCTGTGACTCGATCTCCTTC	
Cytochrome P450, family 7, subfamily A, polypeptide 1	CYP7A1	NM_001205677	Forward	CCAGAAGTTCAGAGAAAGAGACC	138
			Reverse	GCTGGAATGGTGTTTGCTTG	
D site of albumin promoter (albumin D-box) binding protein	DBP	BC108134	Forward	GATACGGTGGAGGTGTTGATG	89
			Reverse	CGAGGGTCAAAGGTCTCATG	
DEAD (Asp-Glu-Ala-Asp) box polypeptide 3, Y-linked	DDX3Y	NM_001172595	Forward	CCAGTTACTTCAGTGATCGAGG	127
			Reverse	TGTCCACTACGCTCAAATCTG	
Dedicator of cytokinesis 6	DOCK6	NM_001192166	Forward	CATCAACAGCCTCACATTCAAG	135
			Reverse	TTCTCCTGGTTCCCAAATGG	
Epiphycan	EPYC	BTU77127	Forward	ACCACATCCCTCTACCACTC	136
			Reverse	CGAATATCCTCTAGTGCCTTACG	
Formiminotransferase cyclodeaminase	FTCD	NM_001205399	Forward	CTGGCATTCAACATCAACCTG	139
			Reverse	TCTCATCCAGGTACCAGCC	
Gamma-aminobutyric acid (GABA) B receptor, 1	GABBR1	BC146241	Forward	GACCATTGAGACTTTTGCCAAG	133
			Reverse	CAGTAGCCCCTTGTAACCATAG	
Guanine nucleotide binding protein (G protein), alpha activating activity polypeptide, olfactory type	GNAL	NM_001102554	Forward	AGGGCGTCGATGAAAAGG	136
			Reverse	ACGATAGTGCTTTTCCCAGAC	
Homeobox D4	HOXD4	NM_001101087	Forward	ATTCCGTGAACCCCAACTAC	139
			Reverse	TGAGCGATTTCAATCCGACG	
HtrA serine peptidase 1	HTRA1	NM_001282082	Forward	CCAGCAAAGCCAAAGAGTTG	124
			Reverse	ACATCGTTTTCCTTGAGCCC	
Insulin-like growth factor binding protein 1	IGFBP1	NM_174554	Forward	AGTAATTATGAGAGCTTGAGGGC	128
			Reverse	AGTTTGTCTCCTGCCTTCTG	
Malic enzyme 2, NAD(+)-dependent, mitochondrial	ME2	BC123669	Forward	CCTGGTGTAATCAAAGCAATGG	119
			Reverse	CTTCCCTCTGTAAGCGTGTATG	
Myosin, heavy chain 1, skeletal muscle, adult	MYH1	NM_174117	Forward	CTGGAAGGAGAAGTTGAGAGTG	137
			Reverse	AAATCCTGGAGCCTGAGAATG	
Nuclear receptor binding protein 2	NRBP2	BC123443	Forward	AGTTCATCCTCTCCTGCCTAG	135
			Reverse	GGTACTGGTGCTGGATGAAG	
Protein phosphatase 1, regulatory subunit 3A	PPP1R3A	NM_001205440	Forward	AATAAGTGTGGAAGGGATGGG	97

			Reverse	CGTATCTGCTCTGATTGACTGG	
RAB3C, member RAS oncogene family	RAB3C	BC112795	Forward	TTTGAGCGTCTCGTGGATATC	98
			Reverse	TCCTTGAGTCTTGTGTTCTGC	
RNA binding motif protein 38	RBM38	NM_001206023	Forward	ACGTGAACTTGGCCTATCTG	137
			Reverse	GATGGCTTGTGGGTAGATGTAG	
Regulator of G-protein signaling 2, 24kDa	RGS2	BC120026	Forward	ACCCCAGAACCAGAAACAAG	129
			Reverse	ATTGAGGCATGGGATACTGTC	
Regulator of G-protein signaling 7	RGS7	NM_174169	Forward	CAGACTATGCGGTTTACCTCTG	126
			Reverse	AAATGAACTCCCACTTCCGG	
Regulator of G-protein signaling 9	RGS9	NM_174170	Forward	ATGAAGACCAGAAACAGACCG	138
			Reverse	CATTGGATGAAAACTGCTCGC	
Sodium channel, voltage gated, type VIII, alpha subunit	SCN8A	NM_001193102	Forward	CTCATCGTGGCTGTCTCTTTAG	137
			Reverse	ACCCTCATCCCTTCAAATCG	
Sodium channel, voltage-gated, type IX, alpha subunit	SCN9A	NM_001110787	Forward	AAAGAGGGACAAAGTGACTACG	137
			Reverse	AAGATGTTCCAGCCTACAGTG	
Solute carrier family 17 (sodium phosphate), member 3	SLC17A3	BC118404	Forward	TGGAAGAGTAGGAACAAAGCG	128
			Reverse	TGCCCTGAAGTATTCGAGTTG	
Solute carrier family 6 (neutral amino acid transporter), member 15	SLC6A15	NM_181023	Forward	CAGAAGCGATGACACATTTCC	76
			Reverse	AGCCCTAGATTTACCAGCATG	
Syntaxin binding protein 5-like	STXBP5L	NM_001206103	Forward	ACCCCAGAACCAGAAACAAG	129
			Reverse	ATTGAGGCATGGGATACTGTC	
Transducin (beta)-like 1X-linked	TBL1X	NM_001145232	Forward	AGTCCGAGGTGTTCATTTGTG	108
			Reverse	CCCCGTTGCTGTTTTCATTC	
Transmembrane protein 59-like	TMEM59L	BC120018	Forward	AGCACCTTAAGTTCCAGCC	135
			Reverse	TCAGGTCCAGTTTCAGCTTG	
Troponin C type 1 (slow)	TNNC1	NM_001034351	Forward	TTTCAGACCTCTTCCGCATG	136
			Reverse	TTGTCACCGTCCTTCATGAG	
Troponin T type 3 (skeletal, fast)	TNNT3	NM_001001441	Forward	GGACAAGCTCAGGGACAAG	110
			Reverse	TGGTGATATCGTATTTCTGGCG	
Uncoupling protein 3 (mitochondrial, proton carrier)	UCP3	NM_174210	Forward	CGACTGTATGCTGAAGATGGTG	128
			Reverse	CCCGTTTCATCTGCTCGTAG	
Ubiquitin specific peptidase 9, Y-linked	USP9Y	NM_001145509	Forward	ATTCTACTCCGTCACAGCAAG	123
			Reverse	AAATACACCCCTCACTTCAGC	
Zinc finger protein 280B	ZNF280B	BC111316	Forward	TTTGGAACTTGAGAGGCTGAG	88
			Reverse	CTGGAAGGGCGTAGGAAAC	

[1]Housekeeping gene.

Table S10. Primer sequences used in real-time PCR analysis of rat tissues

Gene name	Symbol	GenBank ID	5’ → 3’	Sequences	Product size, bp
Adrenomedullin	ADM	NM_012715	Forward	TGATGTTATTGGGTTCGCTCG	124
			Reverse	GACGCTTGTAGTTCCCTCTTC	
Adenosine monophosphate deaminase 1	AMPD1	NM_138876	Forward	CCCTACCCTTACCCAAATCTG	137
			Reverse	ATCTGATGGACCTGGAACTTG	
Apolipoprotein D	APOD	NM_012777	Forward	CTATGCCCTCGTGTATTCCTG	107
			Reverse	GGTAGGTTATTGTTTCTGGAGGG	
Rho guanine nucleotide exchange factor (GEF) 19	ARHGEF19	NM_001108692	Forward	CACCCGTCGATCTAAATGAGG	90
			Reverse	TGGCACTGTTGGTATCTTGAG	
Cation channel, sperm associated 2	CATSPER2	BC079422	Forward	CTCCCAACTCTTCATCCCATG	114
			Reverse	GATCCATATCCATTAGCCCAGG	
Glycoprotein hormones, alpha polypeptide	CGA	NM_053918	Forward	TGTATGGGCTGTTGCTTCTC	127
			Reverse	CTGTGGCCTTAGTAAATGATTTGG	
Collagen, type XXVII, alpha 1	COL27A1	NM_198747	Forward	AGTTTGCTGTTAGTCGGGTC	77
			Reverse	GTGGATTGTGATGTGCTGTG	
Cut-like homeobox 2	CUX2	NM_001271380	Forward	AGAGATTGAGGACCAAAAGCC	128
			Reverse	CATCCACCTCTAAACCCTCTTC	
Cytochrome P450, family 7, subfamily A, polypeptide 1	CYP7A1	NM_012942	Forward	TGAATATCCGCACAGCTAAGG	140
			Reverse	GAAAGTCAAAGGGTCTGGGTAG	
D site of albumin promoter (albumin D-box) binding protein	DBP	BC087668	Forward	CGAGGAACAGAAGGATGAGAAG	137
			Reverse	GGGCGTTTTCCTTCTCCAG	
DEAD (Asp-Glu-Ala-Asp) box polypeptide 3, Y-linked	DDX3Y	FJ775727	Forward	AACAGATTCGGGACTTAGAACG	132
			Reverse	TCCTGTCAGCTTCATCCAAC	
Dedicator of cytokinesis 6	DOCK6	NM_001108997	Forward	AGAGGATGGAAAGTTGGACG	129
			Reverse	CGCTGTGTCTCTGTAGTGATG	
Epiphycan	EPYC	BC161903	Forward	GTAAGGCGCTAGAGGATATTCG	92
			Reverse	AATGGGCAAACGAGGTAGAC	
Formiminotransferase cyclodeaminase	FTCD	AF079233	Forward	AATGTGTCCCCAACTTCTCAG	107
			Reverse	TCCAGCATCAACGTCCAAC	
Gamma-aminobutyric acid (GABA) B receptor, 1	GABBR1	NM_031028	Forward	GCCATCGTGTTCTCTTCCTAC	114
			Reverse	TCCTGTTTTCATGGTGTCCTG	
Guanine nucleotide binding protein (G protein), alpha activating activity polypeptide, olfactory type	GNAL	NM_001191836	Forward	CATCAAGAGCATAGCCCCTATC	122
			Reverse	GCTGGTACTCGTTGGATCTC	
Homeobox D4	HOXD4	NM_001105885	Forward	TGAAATCGCTCACACCCTG	136
			Reverse	GGAGCAGGAAGATGAGGAAG	
HtrA serine peptidase 1	HTRA1	NM_031721	Forward	TCTCCTTCGCAATTCCATCC	86
			Reverse	CTTGGTGACAGTTTTCCCTTTG	
Insulin-like growth factor binding protein 1	IGFBP1	NM_013144	Forward	GAGCCTGTGTACTAGAACCTG	129
			Reverse	GCTGTTCCTCTGTCATCTCTG	
Malic enzyme 2, NAD(+)-dependent, mitochondrial	ME2	NM_001107376	Forward	ACTCCTTGCACTTTGACCTG	96
			Reverse	TGTAAACGCCATCCCCTTG	
Myosin, heavy chain 1, skeletal muscle, adult	MYH1	NM_001135158	Forward	CAATGAGACTAAGACGCCTGG	140
			Reverse	ATCTCTGCTTGAAGTCTGCG	
Nuclear receptor binding protein 2	NRBP2	NM_001135007	Forward	TTTCATCCAGCACCAGTACC	139
			Reverse	GAAGGAAACCTCTGAGTACCG	
Protein phosphatase 1, regulatory subunit 3A	PPP1R3A	NM_001109222	Forward	TAGACGATAATGCCAACCCAG	72

			Reverse	CACCTGCCCTTAGTTGATCAG	
RAB3C, member RAS oncogene family	RAB3C	NM_133536	Forward	TCCTTCACGTCTGCCTTTG	126
			Reverse	GATGGTCCTGTATCTTTCCTGG	
RNA binding motif protein 38	RBM38	NM_001108965	Forward	TGACTCCCCACTACATCTACC	102
			Reverse	GTGTATACTCAAGGTAGGGCG	
Regulator of G-protein signaling 2, 24kDa	RGS2	AY043246	Forward	GTACCCATGGACAAGAGCG	109
			Reverse	GCAAGAAATAGCTCAAACGGG	
Regulator of G-protein signaling 7	RGS7	NM_019343	Forward	CATCCGAAGTCACAGTCCC	78
			Reverse	ATCTGTCGGTGCAACTCATC	
Regulator of G-protein signaling 9	RGS9	AF038006	Forward	CTGTGAGGATCTGAAGTATGGG	110
			Reverse	TGCCGTCTATGTTGATCCATC	
Sodium channel, voltage gated, type VIII, alpha subunit	SCN8A	NM_019266	Forward	TGTCTTGGTCATCTTCGTGG	87
			Reverse	GCCTGGTTCTGTTCCTCATAAG	
Sodium channel, voltage-gated, type IX, alpha subunit	SCN9A	NM_133289	Forward	GGGTCACGATTTCCTACATCTC	81
			Reverse	CCATCGCACATTTCCACTAAC	
Solute carrier family 17 (sodium phosphate), member 3	SLC17A3	BC078710	Forward	GTATAACTGGAGTCCTCAAACCC	133
			Reverse	CAACAGAGCAATTCCAACCAC	
Solute carrier family 6 (neutral amino acid transporter), member 15	SLC6A15	NM_172321	Forward	GTGCTGGCTATACTTCCTGTC	127
			Reverse	AGTTCACGGGTTCTTTCAGG	
Syntaxin binding protein 5-like	STXBP5L	NM_001271250	Forward	CTCATCTCCGAGTGTTTCCAG	114
			Reverse	TGTTCTCGTTCCAATAGCCAG	
Transducin (beta)-like 1X-linked	TBL1X	NM_001106964	Forward	GCTGCCCTAATCTCCATTCTT	83
			Reverse	TCAAATACCGTGCCATCCTC	
Transmembrane protein 59-like	TMEM59L	NM_001271055	Forward	CTGAGTCCCCGAATAACAAGG	116
			Reverse	TTCTGTCTCTGTGGCATTGG	
Troponin C type 1 (slow)	TNNC1	NM_001034105	Forward	GATCTCTTCCGCATGTTTGAC	136
			Reverse	TGTTCTTGTCACCGTCCTTC	
Troponin T type 3 (skeletal, fast)	TNNT3	DQ273678	Forward	GAGGAACAGTACGAAGAGGAAG	116
			Reverse	TTAGGAGCAGTAAGTTTGGGTC	
Uncoupling protein 3 (mitochondrial, proton carrier)	UCP3	BC072546	Forward	GCCCAACATCACAAGAAACG	125
			Reverse	AGGCAGAGACAAAGTGACAG	
Ubiquitin specific peptidase 9, Y-linked	USP9Y	JF827152	Forward	AAAAGTGGACCTGGGCTG	87
			Reverse	AGTTGCTGTAAGTGTAGTGAGG	
Zinc finger protein 280B	ZNF280B	NM_001106384	Forward	CTCCCAAGAACCCATCCAAG	85
			Reverse	CTCCTTTACATTCTCCACCCC	

[1]Housekeeping gene.

Table S11. The significant gene set from DAVID enrichment test with DEGs from fat tissue (FDR < 0.2) and several gene-set databases such as gene ontology and KEGG.

Category	Term	P-value	Benjamini
GOTERM_BP_FAT	acrosome reaction	6.80E-03	6.10E-01
GOTERM_BP_FAT	calcium ion-dependent exocytosis	1.90E-02	7.20E-01
GOTERM_BP_FAT	single fertilization	4.50E-02	8.80E-01
GOTERM_BP_FAT	fertilization	5.70E-02	8.60E-01
GOTERM_BP_FAT	exocytosis	9.00E-02	9.20E-01
GOTERM_BP_FAT	reproductive cellular process	9.60E-02	9.00E-01
GOTERM_BP_FAT	regulation of membrane potential	9.60E-02	9.00E-01
GOTERM_CC_FAT	integral to plasma membrane	6.40E-03	2.40E-01
GOTERM_CC_FAT	intrinsic to plasma membrane	7.20E-03	1.40E-01
GOTERM_CC_FAT	plasma membrane part	4.80E-02	5.00E-01
GOTERM_CC_FAT	plasma membrane	7.40E-02	5.60E-01

Table S12. The significant gene set from DAVID enrichment test with DEGs from liver tissue (FDR < 0.2) and several gene-set databases such as gene ontology and KEGG.

Category	Term	P-value	Benjamini
KEGG_PATHWAY	Sulfur metabolism	6.70E-02	9.80E-01
KEGG_PATHWAY	Hypertrophic cardiomyopathy (HCM)	8.70E-02	9.30E-01
KEGG_PATHWAY	Dilated cardiomyopathy	9.80E-02	8.60E-01
GOTERM_BP_FAT	defense response to bacterium	8.00E-03	1.00E+00
GOTERM_BP_FAT	negative regulation of catalytic activity	1.90E-02	1.00E+00
GOTERM_BP_FAT	detection of external stimulus	2.00E-02	9.90E-01
GOTERM_BP_FAT	regulation of transcription, DNA-dependent	2.20E-02	9.80E-01
GOTERM_BP_FAT	phototransduction, visible light	2.20E-02	9.50E-01
GOTERM_BP_FAT	regulation of RNA metabolic process	2.50E-02	9.40E-01
GOTERM_BP_FAT	defense response	2.60E-02	9.10E-01
GOTERM_BP_FAT	response to bacterium	2.80E-02	9.10E-01
GOTERM_BP_FAT	negative regulation of growth	3.10E-02	9.00E-01
GOTERM_BP_FAT	negative regulation of molecular function	3.40E-02	9.00E-01
GOTERM_BP_FAT	detection of stimulus	3.60E-02	8.90E-01
GOTERM_BP_FAT	ion transport	3.70E-02	8.70E-01
GOTERM_BP_FAT	detection of visible light	4.40E-02	9.00E-01
GOTERM_BP_FAT	second-messenger-mediated signaling	4.80E-02	9.00E-01
GOTERM_BP_FAT	cation transport	5.10E-02	9.00E-01
GOTERM_BP_FAT	phosphate metabolic process	5.10E-02	8.90E-01
GOTERM_BP_FAT	phosphorus metabolic process	5.10E-02	8.90E-01
GOTERM_BP_FAT	regulation of cell cycle	5.40E-02	8.80E-01
GOTERM_BP_FAT	transcription	7.50E-02	9.40E-01
GOTERM_BP_FAT	sensory perception	7.90E-02	9.40E-01
GOTERM_BP_FAT	regulation of growth	8.10E-02	9.40E-01
GOTERM_BP_FAT	regulation of transcription	9.10E-02	9.50E-01
GOTERM_BP_FAT	phosphorylation	9.50E-02	9.50E-01
GOTERM_MF_FAT	sequence-specific DNA binding	4.30E-02	1.00E+00
GOTERM_MF_FAT	inorganic cation transmembrane transporter activity	5.50E-02	1.00E+00
GOTERM_MF_FAT	transcription factor activity	7.50E-02	1.00E+00

Table S13. The significant gene set from DAVID enrichment test with DEGs from muscle tissue (FDR < 0.2) and several gene-set databases such as gene ontology and KEGG.

Category	Term	P-value	Benjamini
KEGG_PATHWAY	Complement and coagulation cascades	2.10E-05	2.60E-03
KEGG_PATHWAY	Calcium signaling pathway	5.50E-04	3.30E-02
KEGG_PATHWAY	Regulation of actin cytoskeleton	1.60E-02	4.80E-01
KEGG_PATHWAY	Chemokine signaling pathway	2.00E-02	4.50E-01
KEGG_PATHWAY	Gap junction	2.20E-02	4.10E-01
KEGG_PATHWAY	Cell adhesion molecules (CAMs)	2.60E-02	4.20E-01
KEGG_PATHWAY	Leukocyte transendothelial migration	3.50E-02	4.60E-01
KEGG_PATHWAY	Intestinal immune network for IgA production	4.00E-02	4.70E-01
KEGG_PATHWAY	Dilated cardiomyopathy	6.20E-02	5.80E-01
KEGG_PATHWAY	Hematopoietic cell lineage	6.40E-02	5.60E-01
KEGG_PATHWAY	ABC transporters	8.70E-02	6.40E-01
KEGG_PATHWAY	Fc gamma R-mediated phagocytosis	8.90E-02	6.10E-01
GOTERM_BP_FAT	immune response	2.10E-06	3.00E-03
GOTERM_BP_FAT	biological adhesion	1.40E-04	9.20E-02
GOTERM_BP_FAT	cell adhesion	1.40E-04	9.20E-02
GOTERM_BP_FAT	endocytosis	9.20E-04	3.50E-01
GOTERM_BP_FAT	membrane invagination	9.20E-04	3.50E-01
GOTERM_BP_FAT	vesicle-mediated transport	9.70E-04	2.80E-01
GOTERM_BP_FAT	secretion	1.10E-03	2.50E-01
GOTERM_BP_FAT	response to wounding	2.00E-03	3.80E-01
GOTERM_BP_FAT	positive regulation of immune system process	2.30E-03	3.70E-01
GOTERM_BP_FAT	positive regulation of response to stimulus	2.90E-03	3.90E-01
GOTERM_BP_FAT	positive regulation of immune response	3.80E-03	4.40E-01
GOTERM_BP_FAT	innate immune response	4.90E-03	5.00E-01
GOTERM_BP_FAT	inflammatory response	5.70E-03	5.10E-01
GOTERM_BP_FAT	spinal cord motor neuron differentiation	6.40E-03	5.20E-01
GOTERM_BP_FAT	membrane organization	7.20E-03	5.30E-01
GOTERM_BP_FAT	rhythmic process	9.10E-03	6.00E-01
GOTERM_BP_FAT	ventral spinal cord development	9.50E-03	5.80E-01
GOTERM_BP_FAT	immune effector process	9.60E-03	5.70E-01
GOTERM_BP_FAT	enzyme linked receptor protein signaling pathway	9.90E-03	5.60E-01
GOTERM_BP_FAT	secretion by cell	1.10E-02	5.80E-01
GOTERM_BP_FAT	extracellular structure organization	1.20E-02	5.80E-01
GOTERM_BP_FAT	blood vessel development	1.20E-02	5.60E-01
GOTERM_BP_FAT	defense response	1.40E-02	5.90E-01
GOTERM_BP_FAT	vasculature development	1.40E-02	5.80E-01

GOTERM_BP_FAT	exocytosis	1.40E-02	5.70E-01
GOTERM_BP_FAT	neuron development	1.50E-02	5.90E-01
GOTERM_BP_FAT	extracellular matrix organization	1.60E-02	5.90E-01
GOTERM_BP_FAT	positive regulation of lymphocyte activation	1.80E-02	6.30E-01
GOTERM_BP_FAT	phagocytosis	2.10E-02	6.60E-01
GOTERM_BP_FAT	cell differentiation in spinal cord	2.20E-02	6.60E-01
GOTERM_BP_FAT	organic acid transport	2.20E-02	6.60E-01
GOTERM_BP_FAT	carboxylic acid transport	2.20E-02	6.60E-01
GOTERM_BP_FAT	development of primary sexual characteristics	2.30E-02	6.60E-01
GOTERM_BP_FAT	positive regulation of leukocyte activation	2.80E-02	7.20E-01
GOTERM_BP_FAT	positive regulation of cell activation	3.20E-02	7.50E-01
GOTERM_BP_FAT	calcium ion-dependent exocytosis	3.20E-02	7.40E-01
GOTERM_BP_FAT	cell junction assembly	3.20E-02	7.40E-01
GOTERM_BP_FAT	spinal cord development	3.20E-02	7.40E-01
GOTERM_BP_FAT	neuromuscular process	3.20E-02	7.40E-01
GOTERM_BP_FAT	transmembrane receptor protein tyrosine kinase signaling pathway	3.30E-02	7.30E-01
GOTERM_BP_FAT	activation of immune response	3.30E-02	7.30E-01
GOTERM_BP_FAT	positive regulation of T cell activation	3.80E-02	7.70E-01
GOTERM_BP_FAT	neuron differentiation	3.90E-02	7.60E-01
GOTERM_BP_FAT	heart morphogenesis	3.90E-02	7.50E-01
GOTERM_BP_FAT	skeletal system morphogenesis	4.10E-02	7.60E-01
GOTERM_BP_FAT	blood vessel morphogenesis	4.40E-02	7.80E-01
GOTERM_BP_FAT	cell activation	4.50E-02	7.80E-01
GOTERM_BP_FAT	regulation of lymphocyte activation	4.80E-02	7.90E-01
GOTERM_BP_FAT	monocarboxylic acid transport	5.00E-02	8.00E-01
GOTERM_BP_FAT	response to yeast	5.10E-02	8.00E-01
GOTERM_BP_FAT	protein maturation	5.50E-02	8.20E-01
GOTERM_BP_FAT	sex differentiation	5.50E-02	8.20E-01
GOTERM_BP_FAT	vesicle docking during exocytosis	5.70E-02	8.20E-01
GOTERM_BP_FAT	response to organic substance	6.00E-02	8.30E-01
GOTERM_BP_FAT	leukocyte activation	6.00E-02	8.30E-01
GOTERM_BP_FAT	cell-cell adhesion	6.30E-02	8.30E-01
GOTERM_BP_FAT	regulation of leukocyte activation	7.10E-02	8.60E-01
GOTERM_BP_FAT	regulation of interleukin-2 production	7.10E-02	8.60E-01
GOTERM_BP_FAT	vesicle docking	7.10E-02	8.60E-01
GOTERM_BP_FAT	cell junction organization	7.10E-02	8.60E-01
GOTERM_BP_FAT	hexose metabolic process	7.20E-02	8.60E-01
GOTERM_BP_FAT	positive regulation of cellular biosynthetic process	7.30E-02	8.60E-01
GOTERM_BP_FAT	protein maturation by peptide bond cleavage	7.60E-02	8.60E-01

GOTERM_BP_FAT	gonad development	7.60E-02	8.60E-01
GOTERM_BP_FAT	regulation of neuron differentiation	7.60E-02	8.60E-01
GOTERM_BP_FAT	endothelial cell development	7.60E-02	8.60E-01
GOTERM_BP_FAT	positive regulation of biosynthetic process	7.80E-02	8.60E-01
GOTERM_BP_FAT	nucleoside monophosphate biosynthetic process	8.10E-02	8.70E-01
GOTERM_BP_FAT	regulation of T cell activation	8.10E-02	8.60E-01
GOTERM_BP_FAT	cell morphogenesis involved in neuron differentiation	8.10E-02	8.60E-01
GOTERM_BP_FAT	regulation of cell activation	8.20E-02	8.60E-01
GOTERM_BP_FAT	regulation of system process	8.20E-02	8.60E-01
GOTERM_BP_FAT	activation of plasma proteins involved in acute inflammatory response	8.70E-02	8.70E-01
GOTERM_BP_FAT	complement activation	8.70E-02	8.70E-01
GOTERM_BP_FAT	reproductive process in a multicellular organism	8.90E-02	8.80E-01
GOTERM_BP_FAT	multicellular organism reproduction	8.90E-02	8.80E-01
GOTERM_BP_FAT	angiogenesis	9.30E-02	8.80E-01
GOTERM_BP_FAT	skeletal system development	9.30E-02	8.80E-01
GOTERM_BP_FAT	cyclic nucleotide biosynthetic process	9.50E-02	8.80E-01
GOTERM_BP_FAT	membrane docking	9.50E-02	8.80E-01
GOTERM_BP_FAT	positive regulation of B cell activation	9.50E-02	8.80E-01
GOTERM_BP_FAT	sexual reproduction	9.70E-02	8.80E-01
GOTERM_CC_FAT	extracellular region part	1.00E-08	2.10E-06
GOTERM_CC_FAT	extracellular region	1.40E-07	1.50E-05
GOTERM_CC_FAT	extracellular matrix	3.20E-06	2.20E-04
GOTERM_CC_FAT	proteinaceous extracellular matrix	4.40E-06	2.30E-04
GOTERM_CC_FAT	extracellular space	1.70E-05	7.20E-04
GOTERM_CC_FAT	plasma membrane	4.30E-03	1.40E-01
GOTERM_CC_FAT	plasma membrane part	9.80E-03	2.50E-01
GOTERM_CC_FAT	extracellular matrix part	1.40E-02	3.00E-01
GOTERM_CC_FAT	interstitial matrix	1.70E-02	3.20E-01
GOTERM_CC_FAT	actin cytoskeleton	2.10E-02	3.50E-01
GOTERM_CC_FAT	troponin complex	5.10E-02	6.30E-01
GOTERM_CC_FAT	vacuole	6.20E-02	6.60E-01
GOTERM_CC_FAT	cell projection	6.50E-02	6.50E-01
GOTERM_CC_FAT	basement membrane	6.60E-02	6.30E-01
GOTERM_CC_FAT	sarcomere	8.50E-02	7.00E-01
GOTERM_CC_FAT	cell soma	8.50E-02	7.00E-01
GOTERM_CC_FAT	intrinsic to plasma membrane	8.70E-02	6.90E-01
GOTERM_CC_FAT	growth cone	9.40E-02	7.00E-01
GOTERM_CC_FAT	site of polarized growth	9.40E-02	7.00E-01
GOTERM_CC_FAT	striated muscle thin filament	1.00E-01	7.00E-01

GOTERM_MF_FAT	calcium ion binding	3.60E-07	1.50E-04
GOTERM_MF_FAT	carbohydrate binding	3.50E-04	7.00E-02
GOTERM_MF_FAT	polysaccharide binding	1.50E-03	1.90E-01
GOTERM_MF_FAT	pattern binding	1.50E-03	1.90E-01
GOTERM_MF_FAT	cation binding	1.90E-03	1.80E-01
GOTERM_MF_FAT	metal ion binding	2.10E-03	1.60E-01
GOTERM_MF_FAT	transcription factor activity	2.50E-03	1.60E-01
GOTERM_MF_FAT	ion binding	2.60E-03	1.40E-01
GOTERM_MF_FAT	copper ion binding	3.50E-03	1.70E-01
GOTERM_MF_FAT	sequence-specific DNA binding	6.30E-03	2.50E-01
GOTERM_MF_FAT	chemokine activity	1.00E-02	3.40E-01
GOTERM_MF_FAT	chemokine receptor binding	1.00E-02	3.40E-01
GOTERM_MF_FAT	cytokine activity	3.00E-02	6.80E-01
GOTERM_MF_FAT	low-density lipoprotein binding	3.50E-02	7.10E-01
GOTERM_MF_FAT	sugar binding	3.80E-02	7.10E-01
GOTERM_MF_FAT	growth factor binding	3.90E-02	6.90E-01
GOTERM_MF_FAT	angiotensin receptor binding	5.00E-02	7.60E-01
GOTERM_MF_FAT	zymosan binding	5.00E-02	7.60E-01
GOTERM_MF_FAT	complement binding	5.00E-02	7.60E-01
GOTERM_MF_FAT	type 1 angiotensin receptor binding	5.00E-02	7.60E-01
GOTERM_MF_FAT	glycosaminoglycan binding	5.20E-02	7.50E-01
GOTERM_MF_FAT	hydrolase activity, acting on carbon-nitrogen (but not peptide) bonds, in cyclic amidines	6.00E-02	7.80E-01
GOTERM_MF_FAT	lipoprotein binding	6.00E-02	7.80E-01
GOTERM_MF_FAT	pancreatic ribonuclease activity	6.70E-02	8.00E-01
GOTERM_MF_FAT	phosphorus-oxygen lyase activity	8.90E-02	8.70E-01
GOTERM_MF_FAT	endoribonuclease activity, producing 3'-phosphomonoesters	8.90E-02	8.70E-01
GOTERM_MF_FAT	transcription regulator activity	9.00E-02	8.60E-01
GOTERM_MF_FAT	RPTP-like protein binding	9.70E-02	8.70E-01
GOTERM_MF_FAT	scavenger receptor activity	9.90E-02	8.60E-01

Table S14. The significant gene set from DAVID enrichment test with DEGs from pituitary-gland tissue (FDR < 0.2) and several gene-set databases such as gene ontology and KEGG.

Category	Term	P-value	Benjamini
KEGG_PATHWAY	Cell adhesion molecules (CAMs)	1.90E-02	8.20E-01
KEGG_PATHWAY	Neuroactive ligand-receptor interaction	2.50E-02	6.60E-01
GOTERM_BP_FAT	muscle organ development	7.00E-04	4.70E-01
GOTERM_BP_FAT	cell wall macromolecule metabolic process	9.50E-04	3.50E-01
GOTERM_BP_FAT	cell wall macromolecule catabolic process	9.50E-04	3.50E-01
GOTERM_BP_FAT	regulation of cell adhesion	1.10E-03	2.90E-01
GOTERM_BP_FAT	striated muscle tissue development	1.30E-03	2.50E-01
GOTERM_BP_FAT	muscle tissue development	1.60E-03	2.50E-01
GOTERM_BP_FAT	hormone transport	1.60E-03	2.20E-01
GOTERM_BP_FAT	tissue morphogenesis	3.20E-03	3.40E-01
GOTERM_BP_FAT	regulation of cell growth	4.50E-03	4.10E-01
GOTERM_BP_FAT	ventricular cardiac muscle morphogenesis	6.50E-03	4.90E-01
GOTERM_BP_FAT	defense response	6.80E-03	4.60E-01
GOTERM_BP_FAT	cytolysis	1.10E-02	6.00E-01
GOTERM_BP_FAT	cardiac muscle tissue morphogenesis	1.10E-02	6.00E-01
GOTERM_BP_FAT	muscle tissue morphogenesis	1.10E-02	6.00E-01
GOTERM_BP_FAT	gonad development	1.20E-02	5.90E-01
GOTERM_BP_FAT	negative regulation of cell adhesion	1.30E-02	6.00E-01
GOTERM_BP_FAT	biological adhesion	1.40E-02	6.10E-01
GOTERM_BP_FAT	cell adhesion	1.40E-02	6.10E-01
GOTERM_BP_FAT	development of primary sexual characteristics	1.50E-02	6.10E-01
GOTERM_BP_FAT	reproductive structure development	1.60E-02	6.10E-01
GOTERM_BP_FAT	regulation of growth	2.70E-02	7.70E-01
GOTERM_BP_FAT	sex differentiation	3.10E-02	8.00E-01
GOTERM_BP_FAT	defense response to bacterium	3.10E-02	8.00E-01
GOTERM_BP_FAT	reproductive developmental process	3.20E-02	7.90E-01
GOTERM_BP_FAT	positive regulation of cellular biosynthetic process	4.20E-02	8.60E-01
GOTERM_BP_FAT	regulation of hormone levels	4.20E-02	8.50E-01
GOTERM_BP_FAT	positive regulation of cell proliferation	4.30E-02	8.40E-01
GOTERM_BP_FAT	positive regulation of biosynthetic process	4.40E-02	8.40E-01
GOTERM_BP_FAT	regulation of cell-substrate adhesion	4.80E-02	8.40E-01
GOTERM_BP_FAT	catecholamine transport	4.90E-02	8.40E-01
GOTERM_BP_FAT	cardiac muscle tissue development	5.10E-02	8.40E-01
GOTERM_BP_FAT	heart morphogenesis	5.10E-02	8.40E-01
GOTERM_BP_FAT	chordate embryonic development	5.20E-02	8.40E-01
GOTERM_BP_FAT	embryonic development ending in birth or egg hatching	5.40E-02	8.30E-01

GOTERM_BP_FAT	endocrine system development	5.40E-02	8.30E-01
GOTERM_BP_FAT	positive regulation of cell adhesion	5.70E-02	8.30E-01
GOTERM_BP_FAT	rhythmic process	6.00E-02	8.40E-01
GOTERM_BP_FAT	thyroid hormone metabolic process	6.10E-02	8.40E-01
GOTERM_BP_FAT	skeletal muscle tissue development	7.40E-02	8.80E-01
GOTERM_BP_FAT	skeletal muscle organ development	7.40E-02	8.80E-01
GOTERM_BP_FAT	regulation of cellular component size	7.60E-02	8.80E-01
GOTERM_BP_FAT	monoamine transport	8.40E-02	9.00E-01
GOTERM_BP_FAT	adrenal gland development	8.40E-02	9.00E-01
GOTERM_BP_FAT	negative regulation of signal transduction	8.60E-02	9.00E-01
GOTERM_BP_FAT	positive regulation of macromolecule metabolic process	8.80E-02	9.00E-01
GOTERM_BP_FAT	cellular amino acid derivative metabolic process	8.80E-02	8.90E-01
GOTERM_BP_FAT	positive regulation of macromolecule biosynthetic process	8.80E-02	8.90E-01
GOTERM_BP_FAT	negative regulation of cell communication	9.50E-02	9.00E-01
GOTERM_BP_FAT	response to bacterium	9.80E-02	9.00E-01
GOTERM_CC_FAT	extracellular region	5.20E-10	7.30E-08
GOTERM_CC_FAT	external side of plasma membrane	7.00E-05	4.90E-03
GOTERM_CC_FAT	extracellular region part	3.40E-04	1.60E-02
GOTERM_CC_FAT	cell surface	3.80E-04	1.30E-02
GOTERM_CC_FAT	plasma membrane	1.90E-03	5.20E-02
GOTERM_CC_FAT	extracellular matrix	1.40E-02	2.80E-01
GOTERM_CC_FAT	plasma membrane part	2.50E-02	4.00E-01
GOTERM_CC_FAT	proteinaceous extracellular matrix	2.90E-02	4.10E-01
GOTERM_CC_FAT	extracellular space	3.70E-02	4.50E-01
GOTERM_CC_FAT	extracellular matrix part	4.40E-02	4.70E-01
GOTERM_CC_FAT	apical part of cell	5.50E-02	5.10E-01
GOTERM_CC_FAT	anchored to membrane	9.00E-02	6.70E-01
GOTERM_CC_FAT	apical plasma membrane	9.80E-02	6.70E-01
GOTERM_MF_FAT	neuropeptide hormone activity	3.70E-03	6.50E-01
GOTERM_MF_FAT	lysozyme activity	8.60E-03	7.00E-01
GOTERM_MF_FAT	identical protein binding	9.50E-03	5.90E-01
GOTERM_MF_FAT	cell surface binding	1.50E-02	6.50E-01
GOTERM_MF_FAT	hormone activity	1.90E-02	6.60E-01
GOTERM_MF_FAT	protein homodimerization activity	2.30E-02	6.60E-01
GOTERM_MF_FAT	extracellular matrix structural constituent	2.50E-02	6.40E-01
GOTERM_MF_FAT	enzyme inhibitor activity	4.60E-02	8.00E-01
GOTERM_MF_FAT	protein dimerization activity	4.60E-02	7.70E-01
GOTERM_MF_FAT	cytoskeletal protein binding	4.60E-02	7.70E-01
GOTERM_MF_FAT	peptidase inhibitor activity	5.90E-02	8.10E-01

GOTERM_MF_FAT	actin binding	8.00E-02	8.80E-01
GOTERM_MF_FAT	transmembrane receptor protein tyrosine kinase activity	1.00E-01	9.10E-01

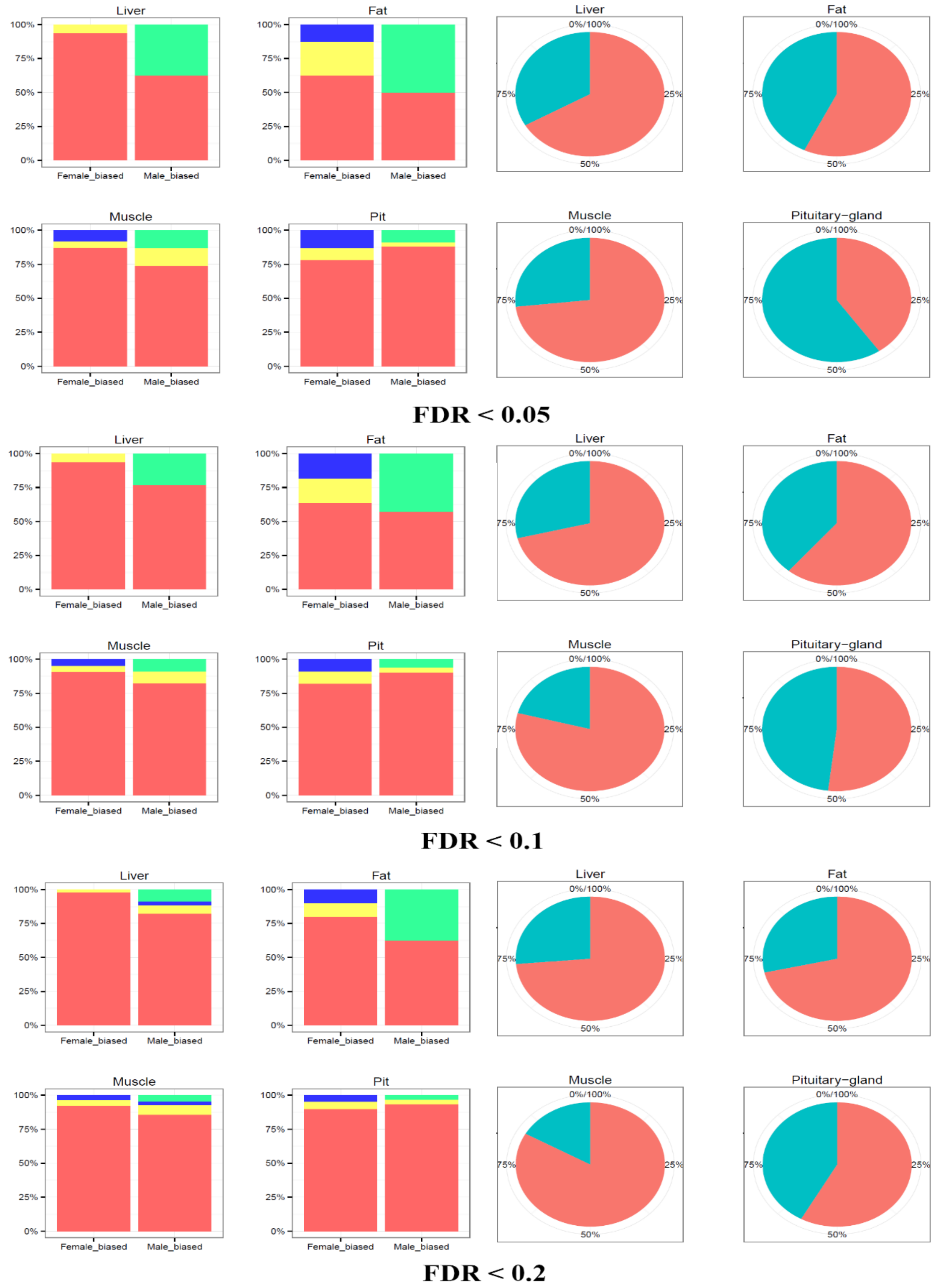

Figure S1. Exploration of gender biases and chromosomal effects on tissues

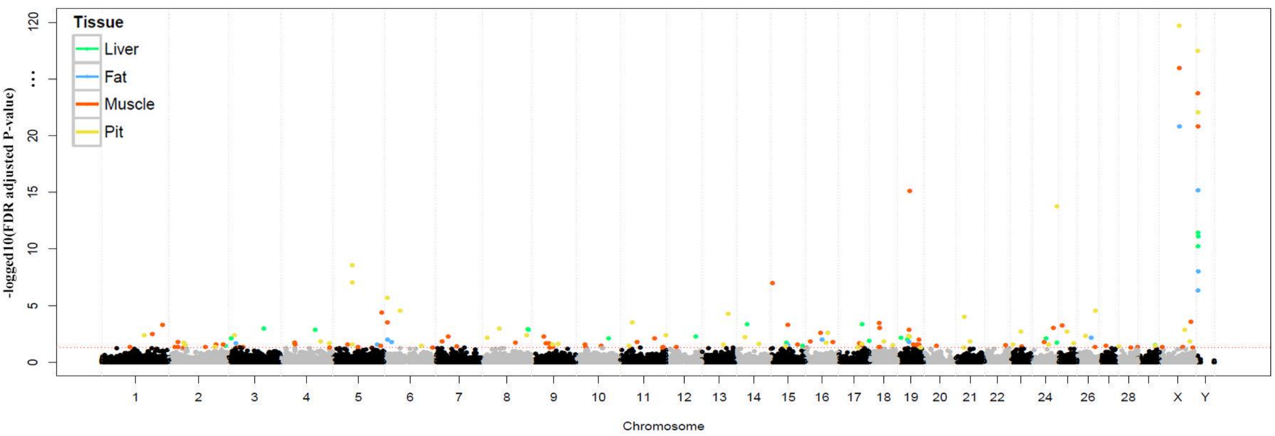

Figure S2. Manhattan plot for allocating sexual dimorphic genes from located chromosomal position with y-axis is a –log10 transformed FDR adjusting P-values

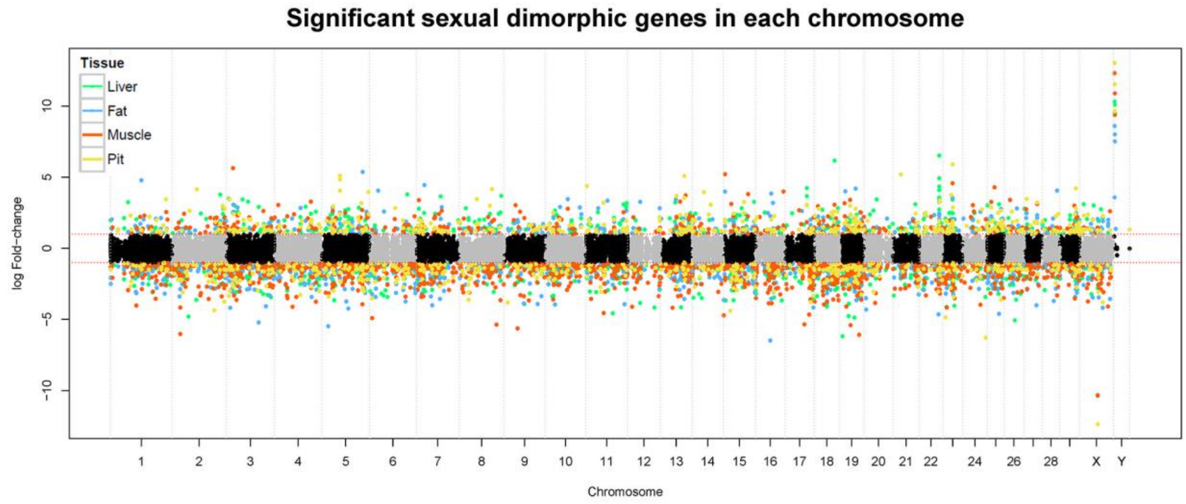

Figure S3. Overlapped Manhattan plot across the all tissues for allocating sexual dimorphic genes from located chromosomal position with y-axis is a log2 fold changes between female and male. Total 13,148 genes were visualized in this plot.

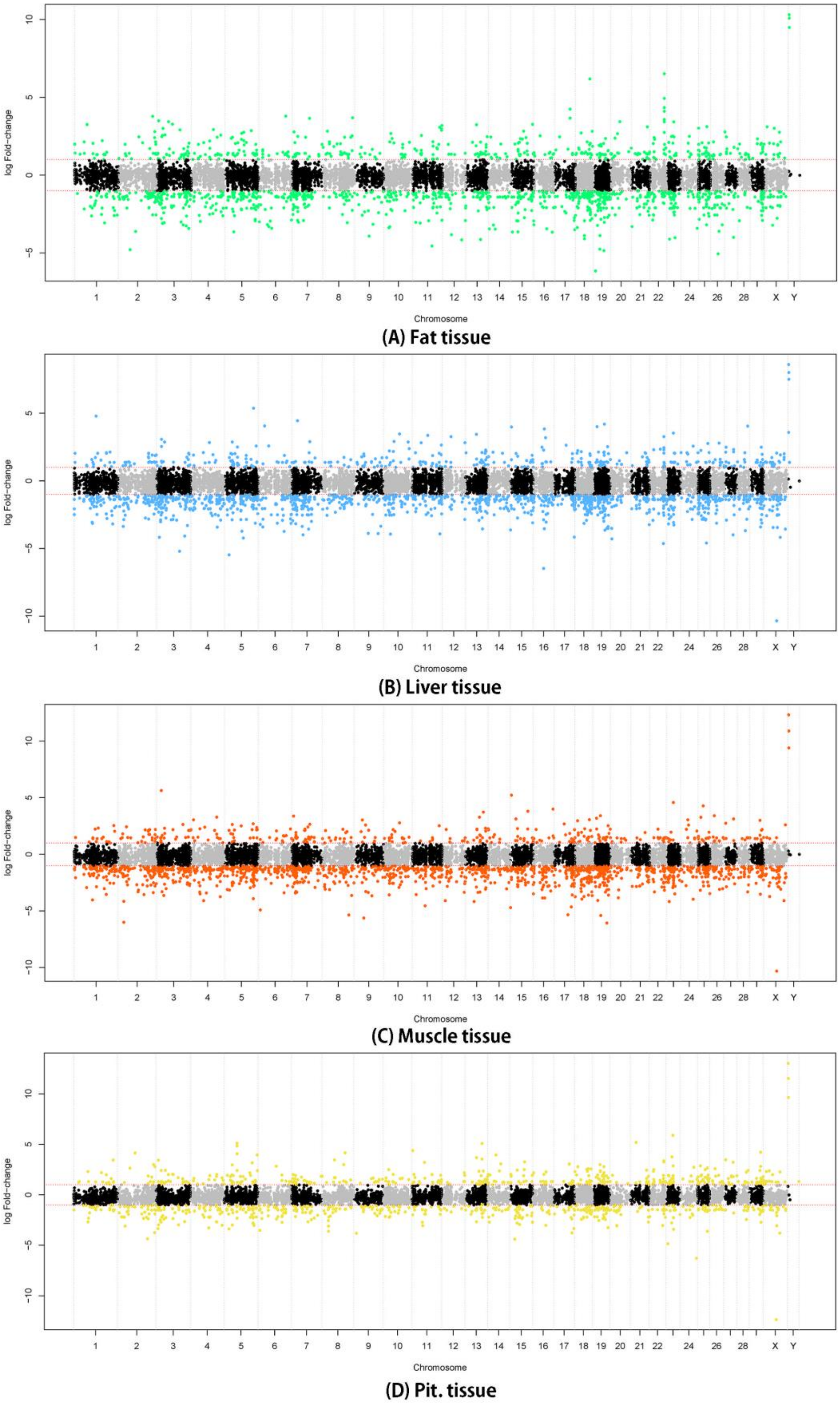

Figure S4. Separated Manhattan plots for significantly observed genes in M1

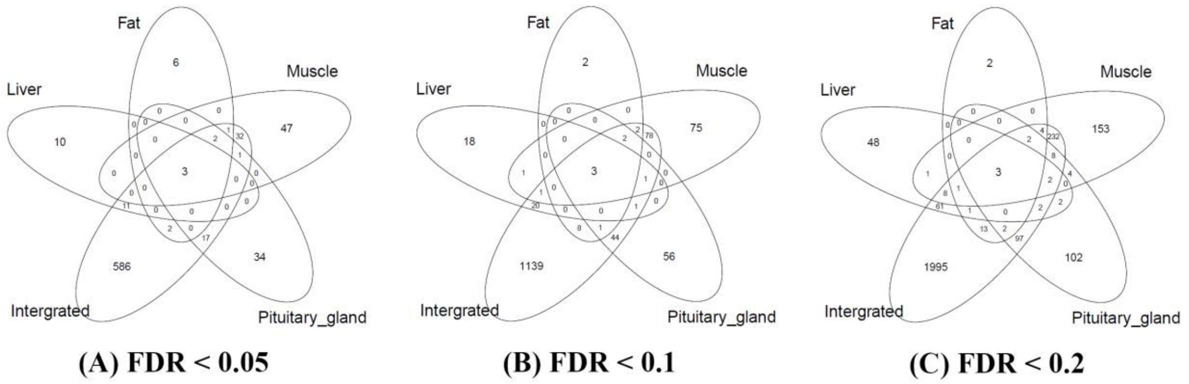

Figure S5. Venn-diagram for M1 (each-tissue model) and M2 (integrated model)

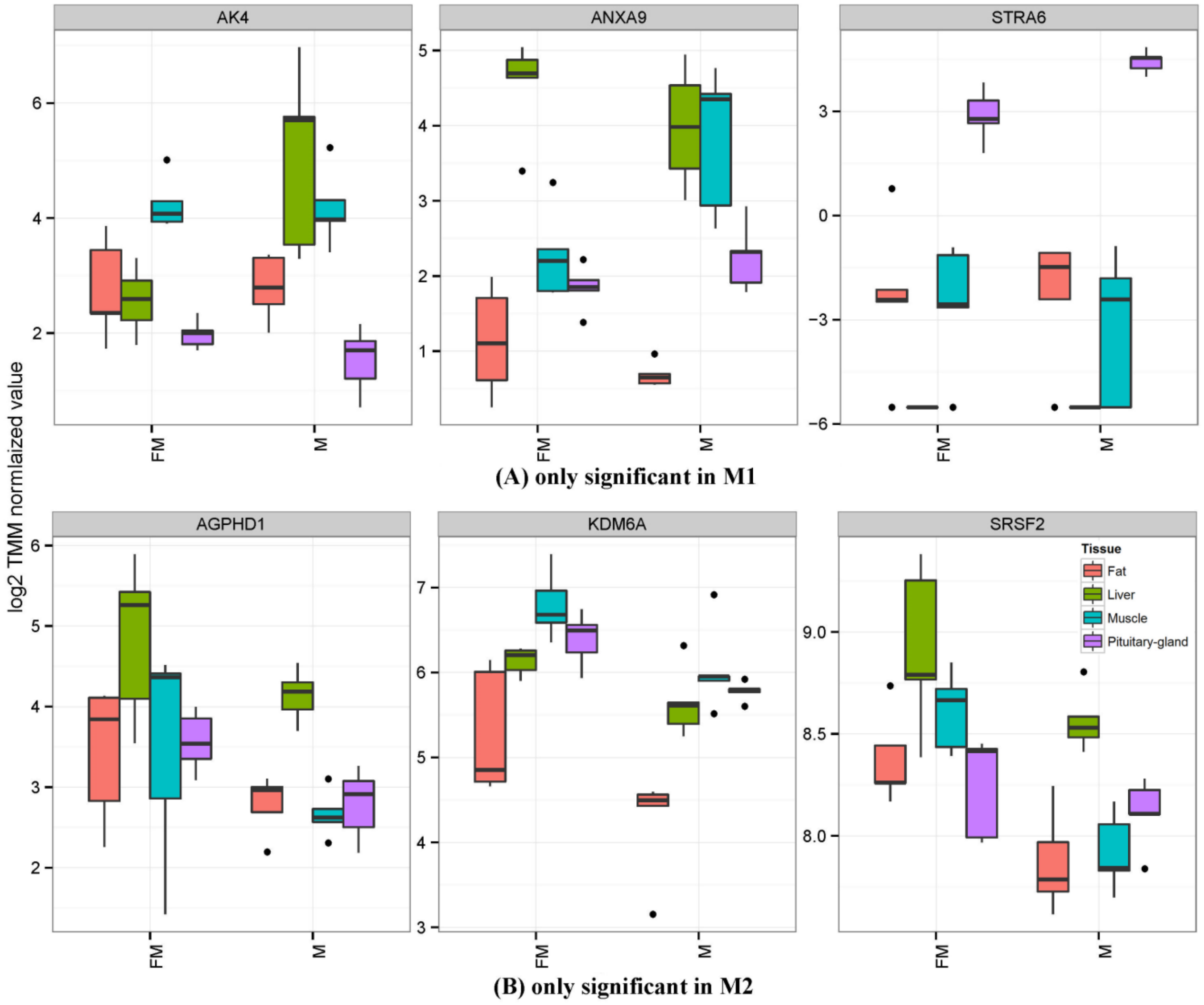

Figure S6. Box plots for comparison between M1 and M2. (A) Representative significant genes in M1 (tissue separately model). (B) Representative significant genes in M2 (Integrated model). Y-axis represent normalized values by TMM normalization using edgeR and X-axis represent gender. Four colored lines were drew in each tissues, which is connected between average of female and male expression values, respectively.

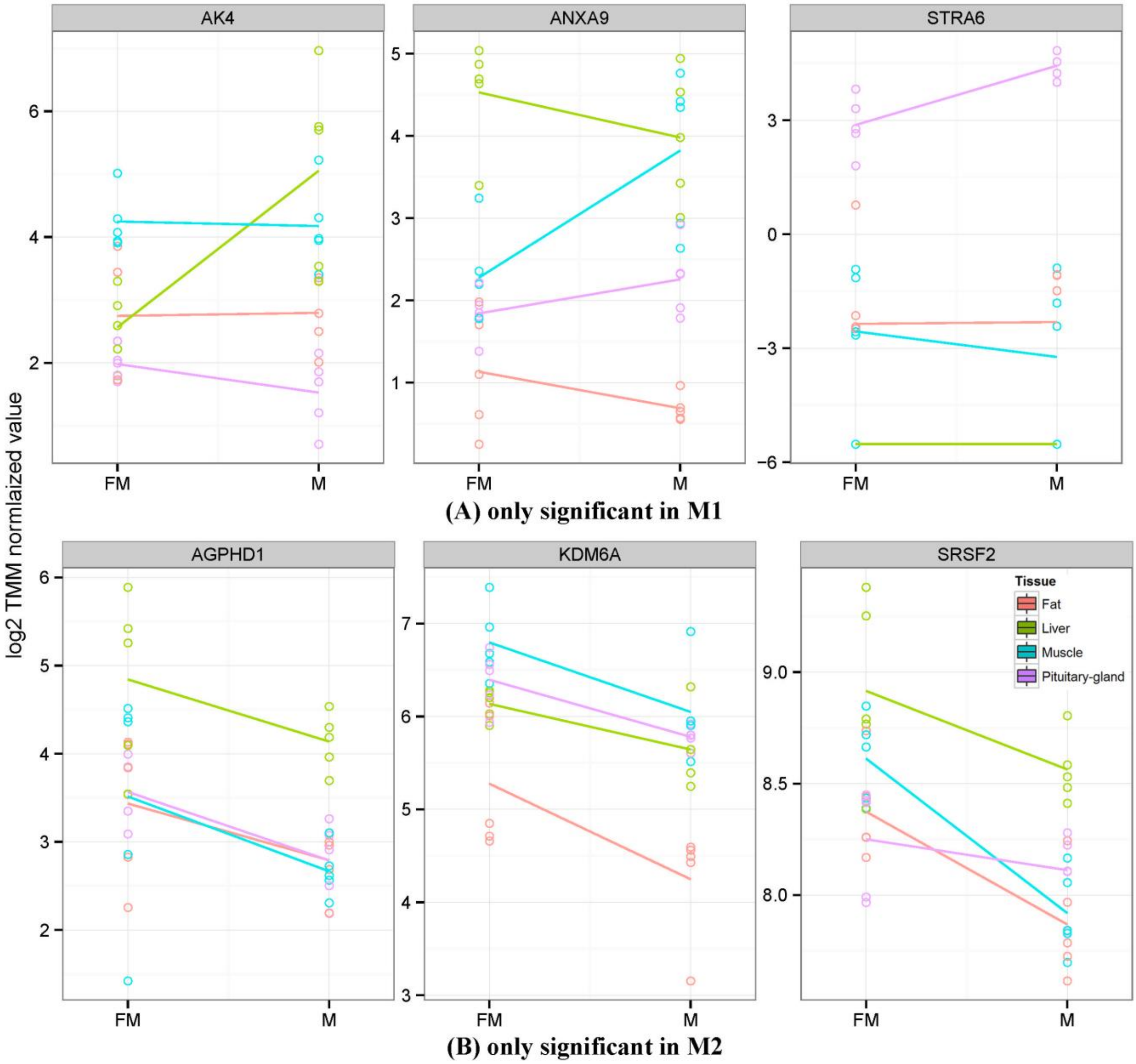

Figure S7. Line plots for comparison between M1 and M2. (A) Representative significant genes in M1 (tissue separately model). (B) Representative significant genes in M2 (Integrated model). Y-axis represent normalized values by TMM normalization using edgeR and X-axis represent gender. Four colored lines were drew in each tissues, which is connected between average of female and male expression values, respectively.

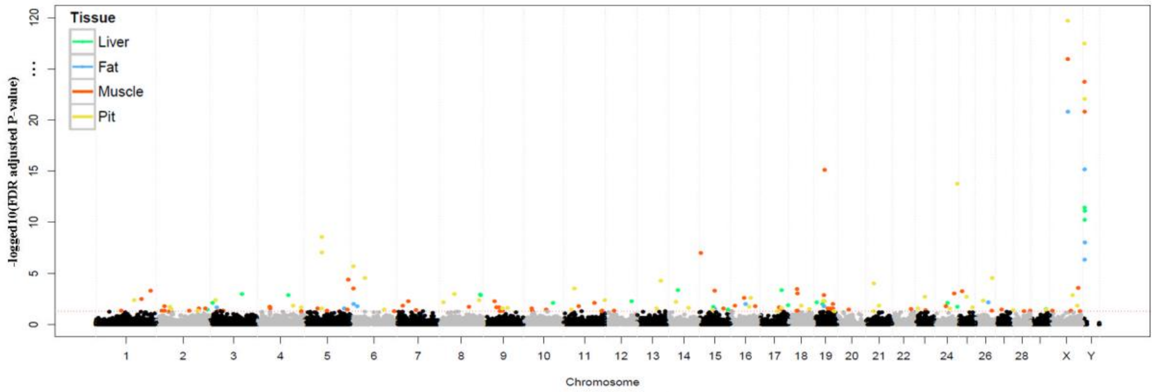

(A) Manhanttan plot from M1

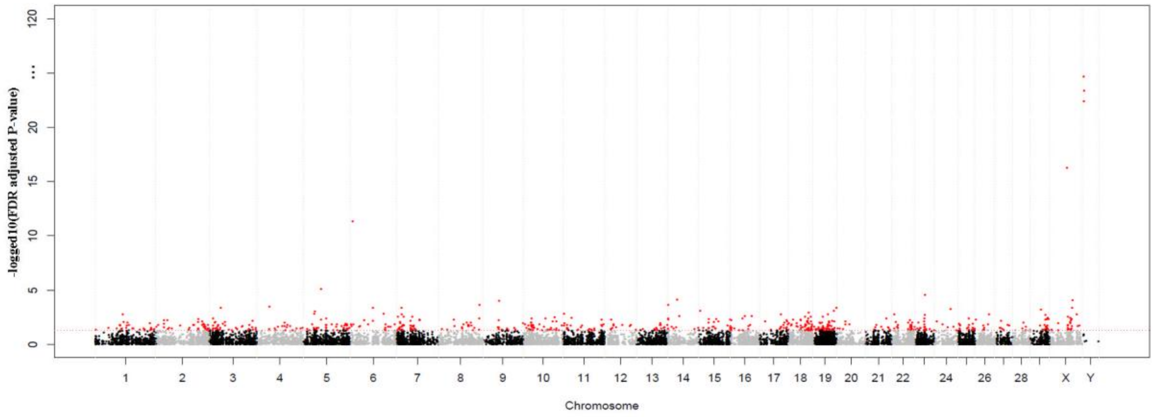

(B) Manhanttan plot from M2

Figure S8. Manhattan plots. (A) FDR adjusted P-value of the M1 method for each sampled tissue and chromosomal location. (B) FDR adjusted P-value of the M2 method. Red marked genes are significant in M2 under FDR < 0.05.

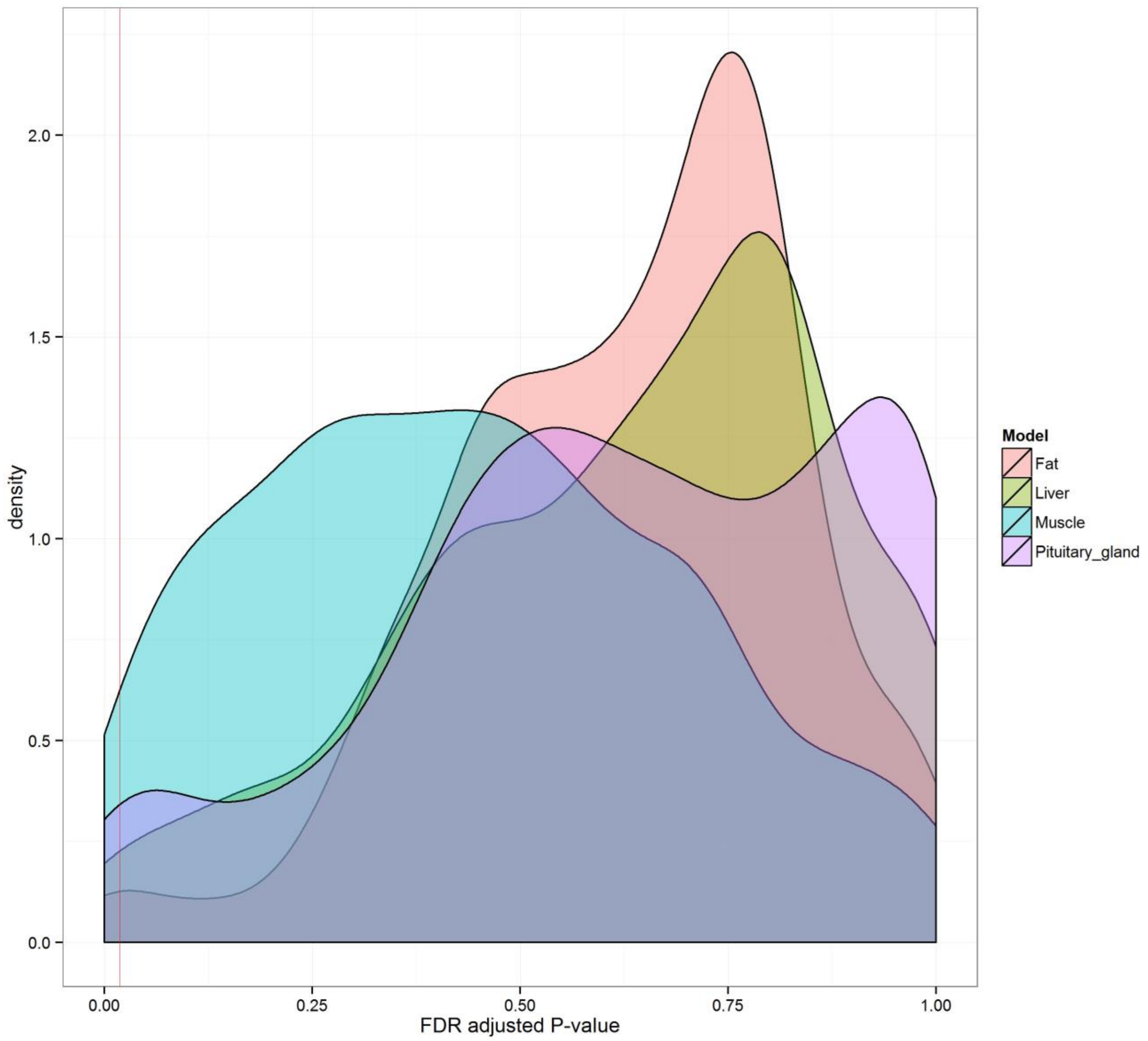

Figure S9. Visualizing densities of the FDR adjusted p-values from M1 for 655 detected DEGs from M2. The red-vertical line represents 5% significance level.

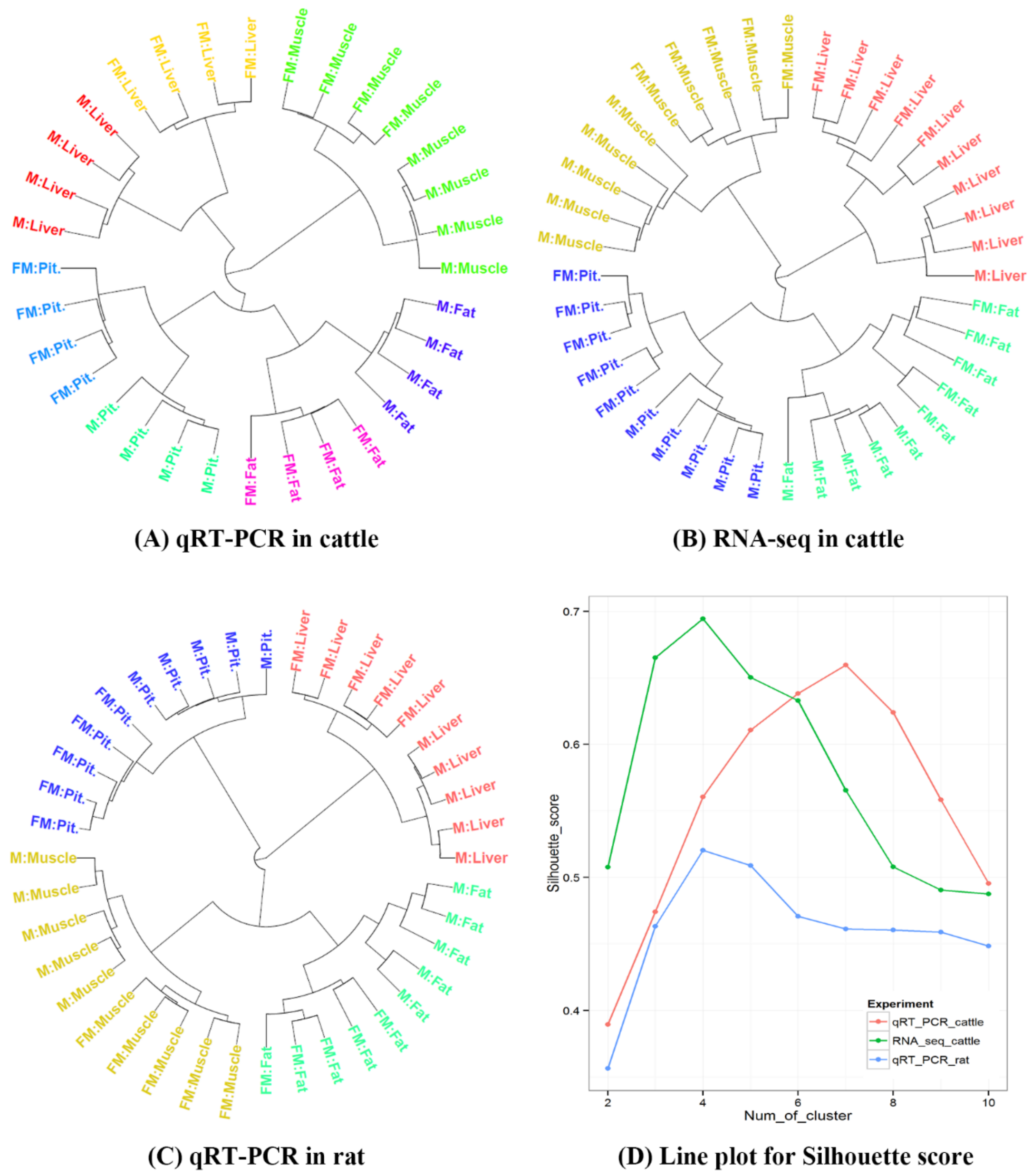

Figure S10. Hierarchical clustering analysis of 40 sexual dimorphic genes for qRT-PCR validation of RNA-seq results. Three trees were visualized with optimal number of clusters based on the Silhouette score. The colors represent estimated groups derived from hierarchical clustering and its optimal number of clusters, respectively. (A) qRT-PCR for cattle species with 32 samples (*k*=7). (B) RNA-seq result for cattle species (*k*=4). (C) qRT-PCR for rat species with 40 samples (*k*=4). (D) Silhouette score plot for detecting optimal number of clusters.

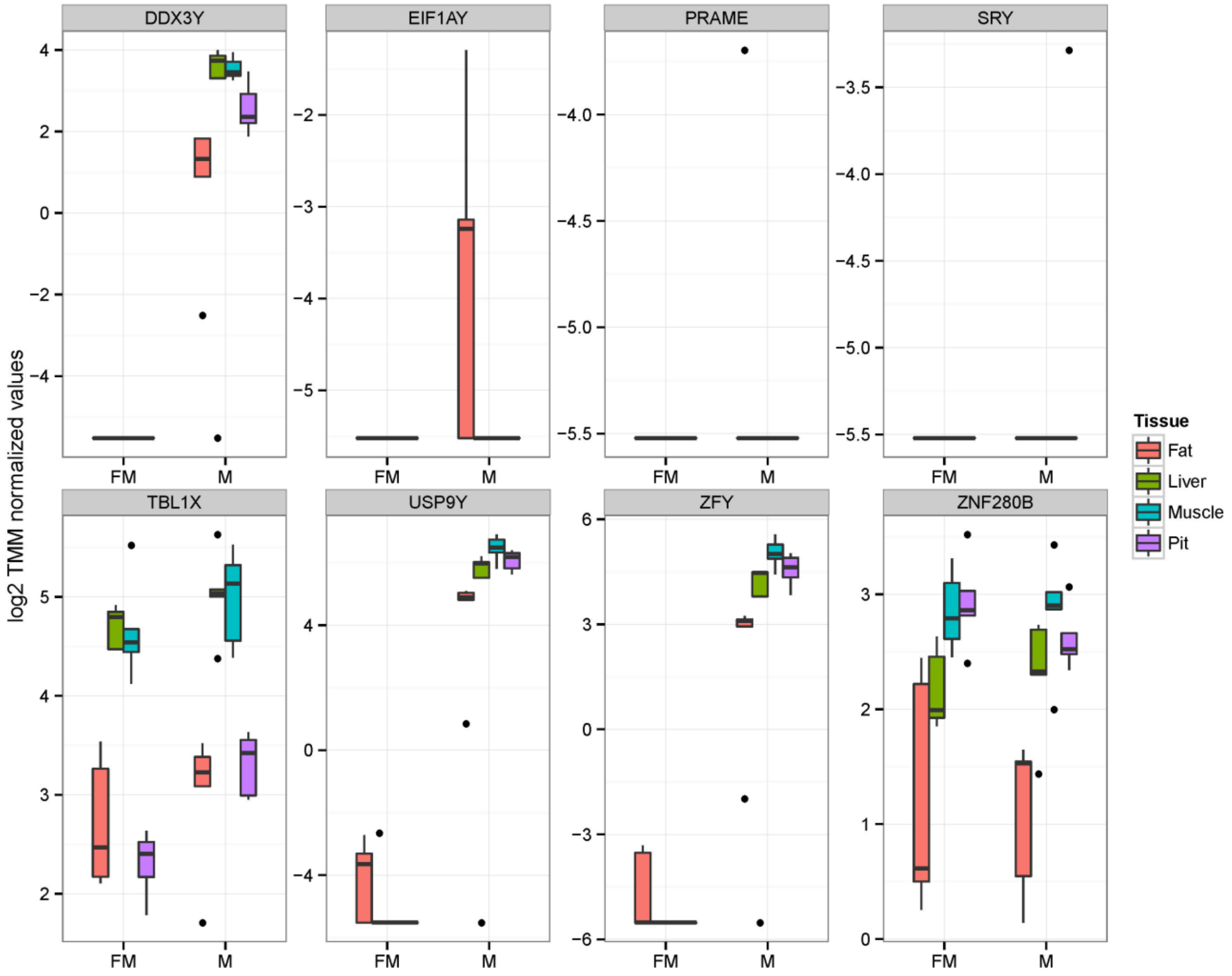

Figure S11. Gene expression patterns for annotating 8 Y-linked genes based on bosTau7 genome.

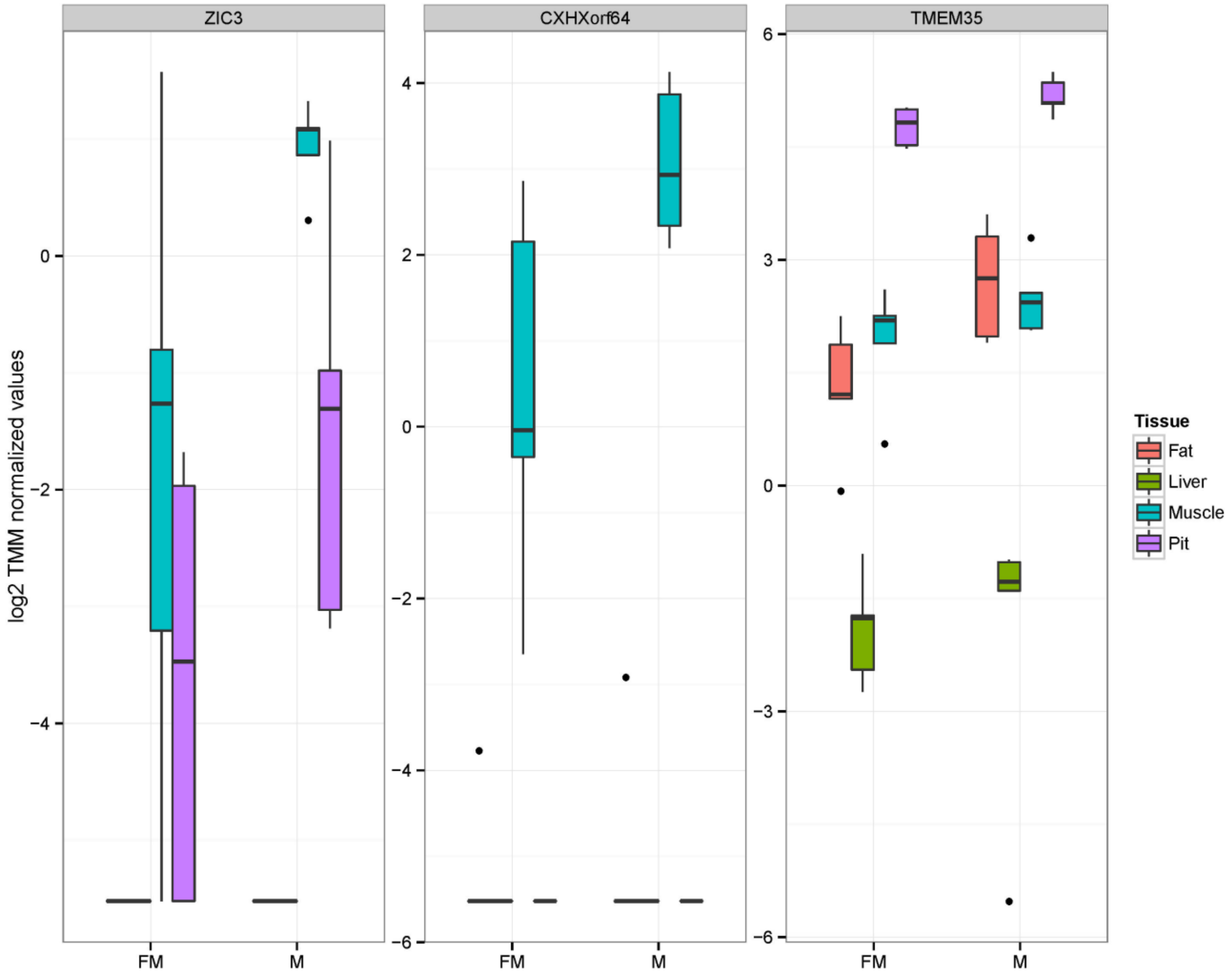

Figure S12. Detected three male-over-expressed genes (FDR adjusted P-value < 0.1)

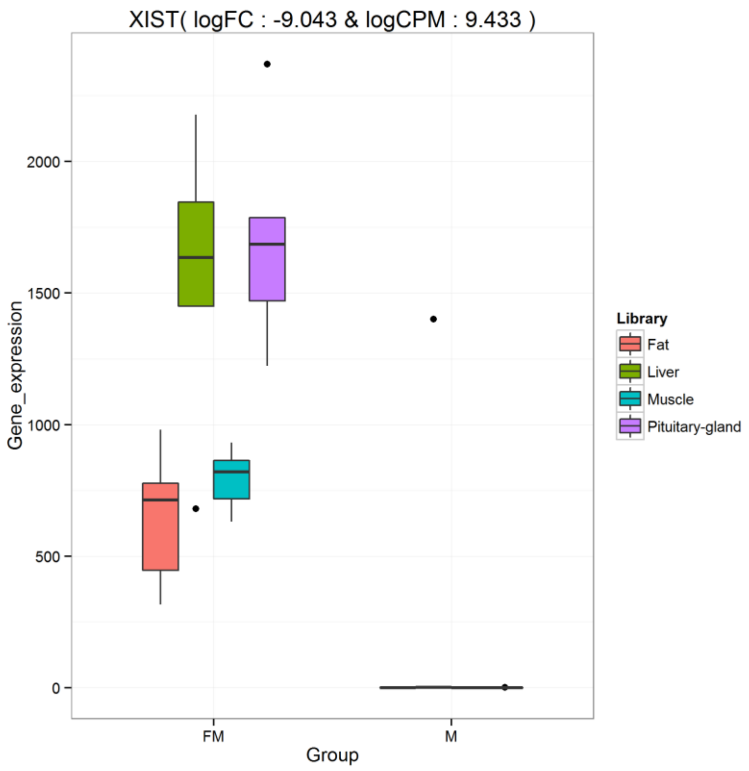

Figure S13. Box-plot describing gene expression of XIST

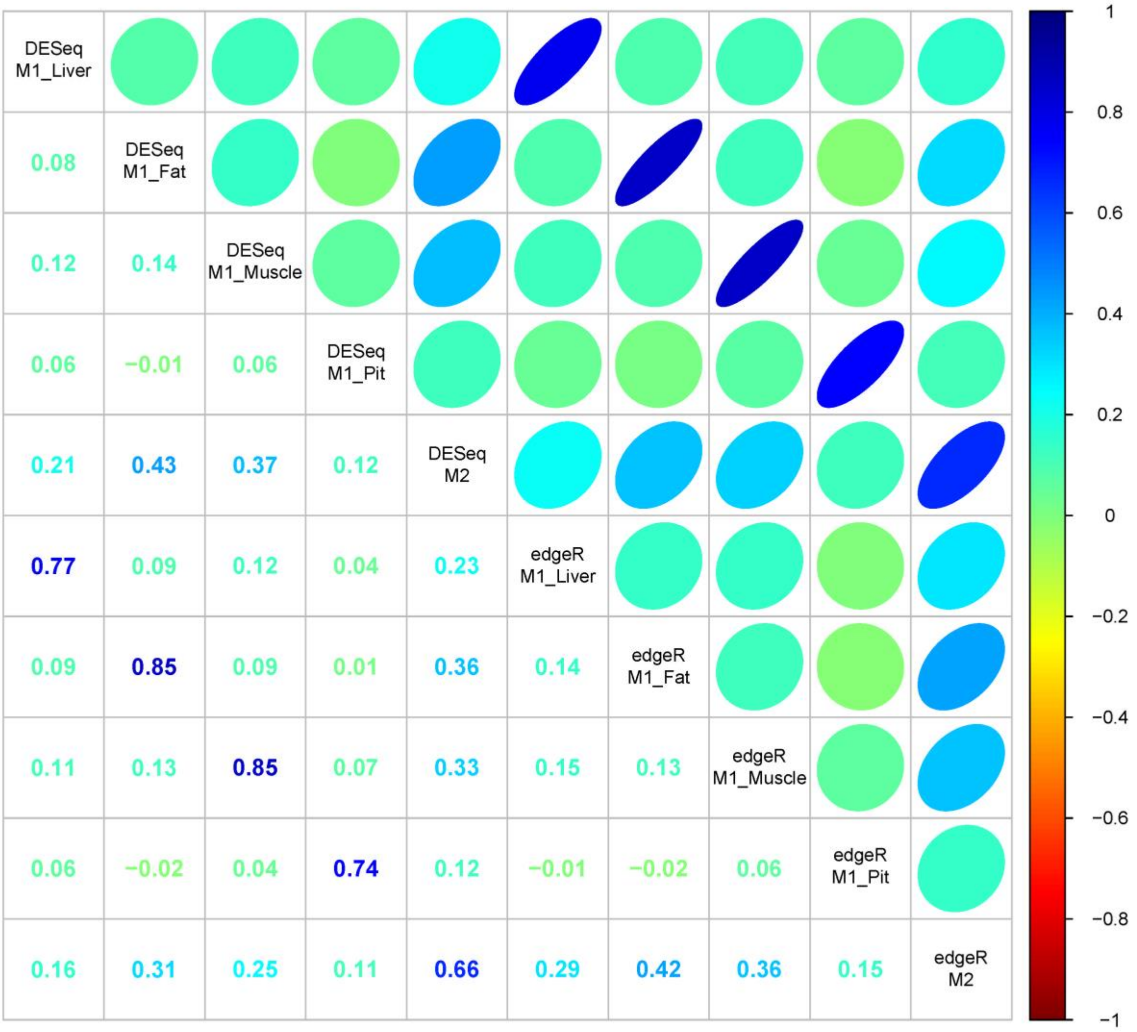

Figure S14. The correlation plot for comparison between two statistical analysis tools such as edgeR and DESeq2. Spearman correlation coefficients were employed with each P-value derived from different hypothesis tests. Higher correlation coefficients (0.66 to 0.85) were observed in same hypothesis tests across the edgeR and DESeq2.

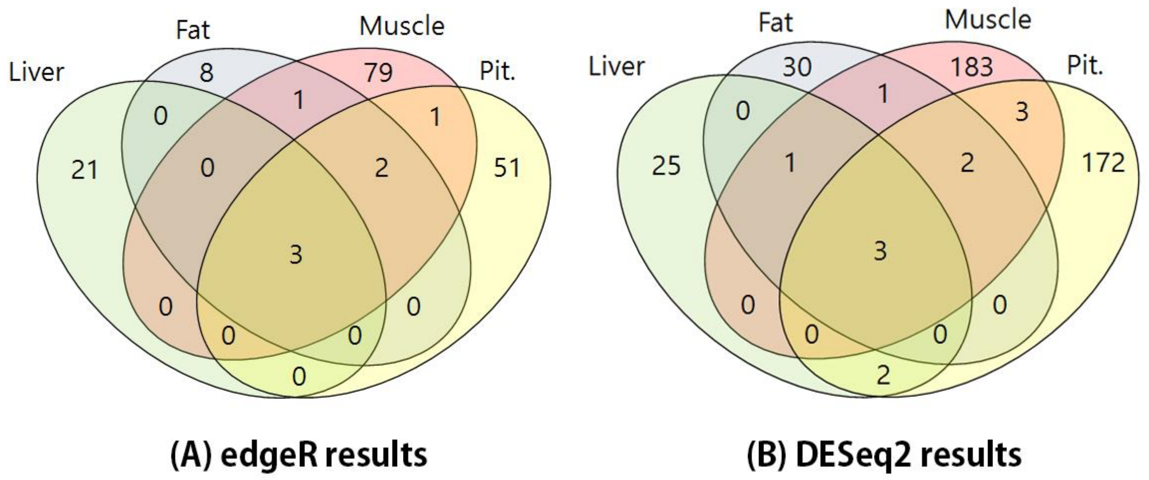

Figure S15. Venn diagram of DEGs list (FDR adjusted P-value < 0.05) for comparison between two statistical analysis tools such as edgeR and DESeq2. In the DESeq2, relatively large number of DEGs were significantly detected compared to the edgeR.

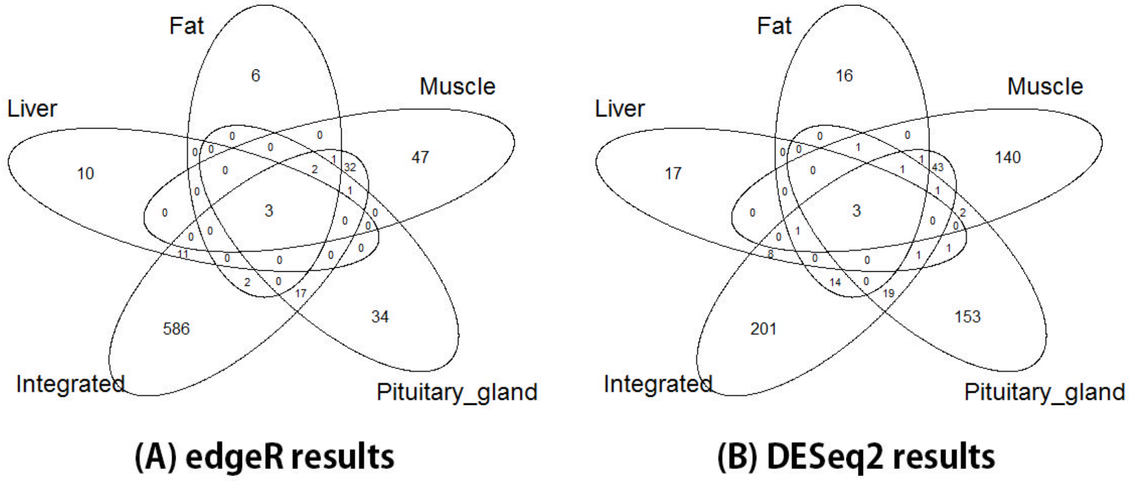

Figure S16. Venn diagram of DEGs list (FDR adjusted P-value < 0.05) for comparison between two statistical analysis tools such as edgeR and DESeq2. The lager number of detected genes in M1 than M2, which were observed in edgeR and DESeq2.

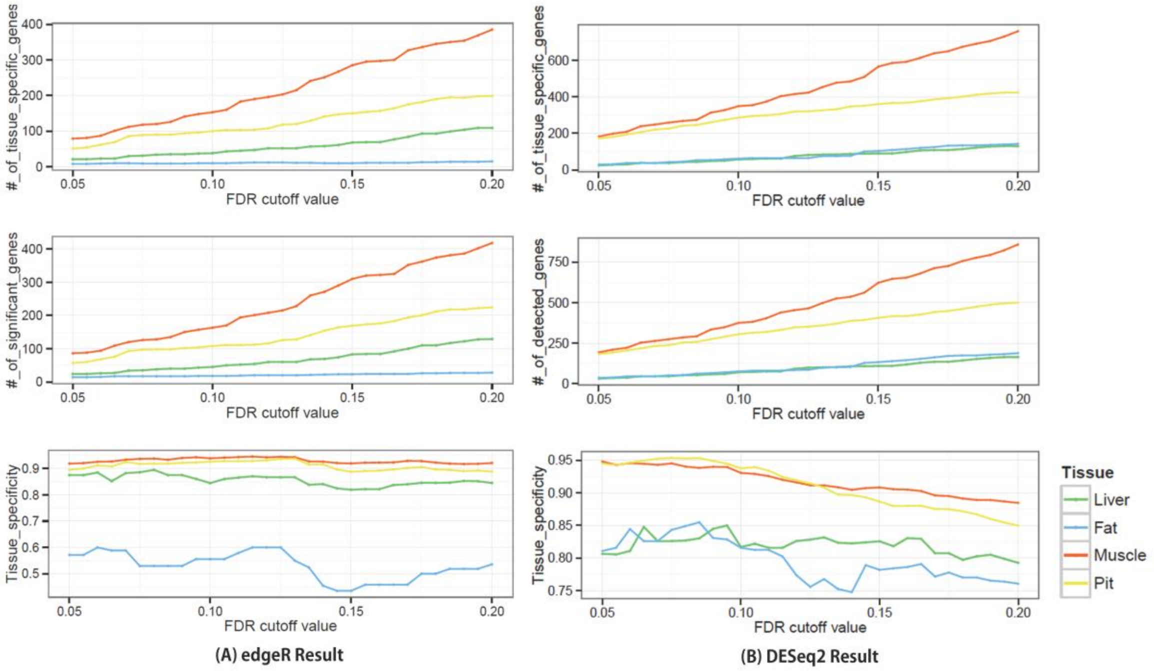

Figure S17. Tissue specificity of the detected DEGs using edgeR and DESeq2, respectively. Number of detected genes and tissue specific genes by FDR cutoff and their tissue specificity calculation. While edgeR and DESeq2 generate almost same result (Relatively Higher tissue specificity of the Muscle and pituitary gland and relatively lower tissue specificity of the liver and fat tissue)

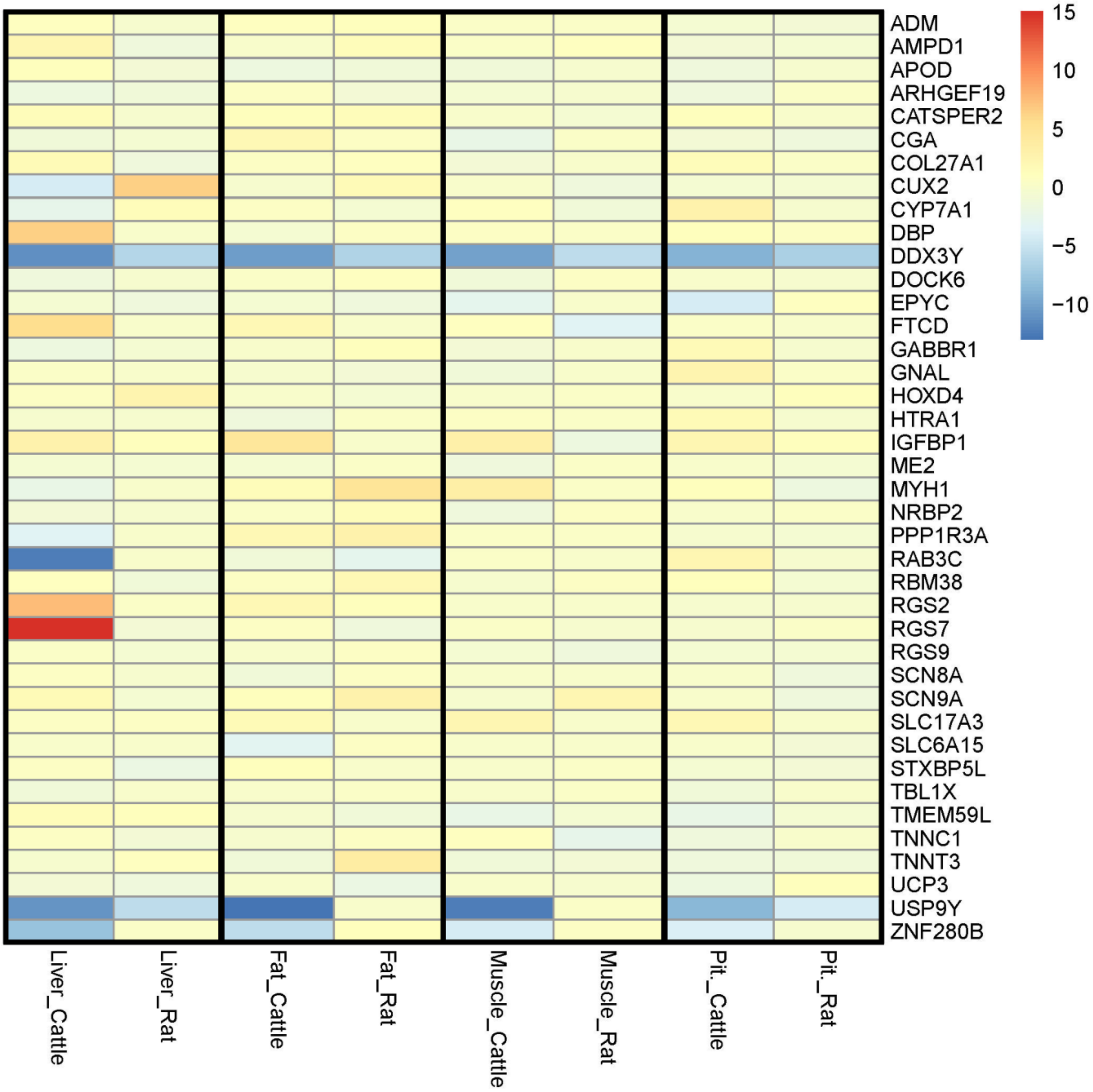

Figure S18. Comparative heatmap between bovine and rat qRT-PCR results. The intensities represent FM:M ratio (Female gene expression / Male gene expression).
